# Practice environment and burnout in intensive care units: is job satisfaction a mediator in this relationship?*

**DOI:** 10.1590/1518-8345.7659.4713

**Published:** 2025-11-03

**Authors:** Jussara Aparecida da Silva Furlan, Edinêis de Brito Guirardello

**Affiliations:** 1 Universidade Estadual de Campinas, Faculdade de Enfermagem, Campinas, SP, Brazil

**Keywords:** Nursing, Professional Burnout, Health Facility Environment, Job Satisfaction, Intensive Care Units, Emotional Exhaustion

## Abstract

to analyze the relationship between the perception of nursing professionals in intensive care units about the practice environment, the burnout level and job satisfaction, based on a theoretical model.

a cross-sectional correlational study was carried out with 114 nursing professionals from a university hospital in the northwest of São Paulo state. A personal and professional characterization form, the Practice Environment Scale*,* the Maslach Burnout Inventory and the job satisfaction subscale of the Safety Attitudes Questionnaire were used. Descriptive analyses were carried out, and comparison tests (non-paired Student’s t-test, Mann-Whitney, ANOVA and Kruskal-Wallis), correlations (Pearson or Spearman) and structural equation modeling were used in order to evaluate the mediating effect.

the professionals reported a favorable practice environment, job satisfaction, low levels of emotional exhaustion, high levels of personal fulfillment and moderate levels of depersonalization. Job satisfaction was found to have a partial mediating effect between the practice environment and burnout.

actions aimed at improving the practice environment can directly reduce burnout or mediate it by increasing job satisfaction. Understanding these relationships is fundamental to promoting a favorable environment, minimizing burnout and increasing job satisfaction.

## Highlights

**(1)** The study confirms the relationship between the practice environment and burnout. **(2)** Job satisfaction partially mediates the relationship between the environment and burnout. **(3)** Improving the environment reduces burnout, both directly and by increasing satisfaction. **(4)** The theoretical model helps managers to identify specific interventions.

## Introduction

The nursing practice environment is understood as the organizational characteristics of the work environment that facilitate or hinder nursing practice^(1)^. In Intensive Care Units (ICUs), this environment is highly specialized and multidimensional, its main purpose being to provide intensive care to seriously ill patients. The nursing professionals who work in these units perform activities aimed at stabilizing and recovering patients and are also subject to emotional impacts resulting from the demands and characteristics of that workplace^(2-3)^.

In recent years, nursing activities have gained prominence due to their role in caring for COVID-19 patients. ICUs have been the environments with the greatest demand for health care, undergoing a process of expanding the number of beds and increasing the need for human and material resources. This practice scenario forced nursing professionals to carry out their activities in the face of scarce resources and work overload^(2)^, in addition to witnessing countless deaths, the illness of colleagues, their own illness and the constant fear of taking the virus home^(4)^, characterizing a practice environment as conducive to professional illness^(3,5)^.

Given this context of overload and adversity faced during the pandemic, it is essential to understand which elements of the work environment contribute to the health and performance of nursing teams. The work environment is considered favorable when it enables the nursing team to carry out their activities autonomously, has adequate human and material resources, has managers who maintain open communication and offer opportunities for professional development. In contrast, an unfavorable environment is characterized by a shortage of supplies, work overload, lack of support from leaders, lack of collaboration between health teams, dissatisfaction and burnout among professionals^(6-7)^.

Health conditions directly related to the work environment are widely described in the literature, including burnout, a frequent problem among nursing professionals. This syndrome is associated with excessive pressure in the workplace, resulting in emotional exhaustion, depersonalization and reduced personal fulfillment^(8)^. Studies show high burnout rates among nurses working in ICUs^(3,8)^_,_ with negative impacts on both patient safety^(9)^ and care quality^(10-11)^.

A systematic review analyzed 85 studies involving 288,581 nurses from 32 countries and identified an average burnout prevalence of 30.7% (±9.7%). Burnout was associated with negative outcomes such as a worse safety climate, higher incidence of patient falls, medication errors, missed care, lower care quality and patient dissatisfaction, regardless of age, gender, professional experience or geographical location^(9)^.

The relationship between burnout and job satisfaction has been studied among nursing professionals working in intensive care units. Burnout and job satisfaction are inversely related. A recent systematic review study, involving 18 studies and a total of 3,168 ICU nurses, identified an overall burnout prevalence of 37.5% and revealed a significant inverse correlation between burnout and job satisfaction, showing that more satisfied professionals have a lower risk of developing the syndrome^(12)^. High levels of emotional exhaustion and depersonalization are associated with lower job satisfaction, directly affecting motivation, commitment and the quality of the care provided. On the other hand, satisfied professionals tend to have lower burnout levels, greater resilience in the face of environmental adversity and a better perception of their own performance^(13)^.

In this context, job satisfaction can be a protective factor against emotional exhaustion and professional burnout. The same meta-analysis showed that satisfied professionals demonstrate greater resilience, better performance and lower rates of absenteeism and turnover^(12)^. The nursing team’s satisfaction does not only refer to the individual well-being of its members and their perception of their professional life, but also to the quality and health of the work environment. Inefficient management of the practice environment, associated with high levels of emotional exhaustion, has been linked to increased job dissatisfaction among nursing professionals^(9)^.

Considering this relationship, when the practice environment, burnout and job satisfaction are analyzed together, it can be seen that positive environments are correlated with lower levels of burnout and lower levels of job dissatisfaction^(7,14)^. An integrated analysis of these aspects allows us to understand the contribution of the practice environment to nursing results and patient outcomes, enabling managers to identify areas in need for improvement^(1)^. This means that an environment considered favorable to nursing practice can contribute to positive repercussions for patients, professionals and organizations^(15)^.

Despite the growing volume of research into the nursing practice environment, there are still few studies that investigate the relationship between the practice environment, burnout and job satisfaction among nursing professionals in an integrated way. The literature shows advances in the individual understanding of these elements, but there is a gap in the joint analysis of those factors and the proposal of theoretical models to explain their interactions.

Given the importance of the practice environment in the professional burnout and satisfaction of ICU nursing professionals, this study is based on the following guiding question: does job satisfaction mediate the relationship between the practice environment and burnout in ICU nursing professionals? Therefore, the aim of this study was to analyze the relationship between the perception of nursing professionals in intensive care units about the practice environment, the burnout level and job satisfaction, based on a theoretical model.

## Method

### Type

Cross-sectional and correlational study which followed the recommendations of the Strengthening the Reporting of Observational Studies in Epidemiology (STROBE).

### Site

This study was conducted in adult intensive care units at a university hospital in the city of Ribeirão Preto, SP, Brazil. The hospital was a reference for critical cases of COVID-19 in its catchment area and, for this reason, it underwent an expansion in the number of intensive care beds. At the beginning of data collection, it had 84 intensive care beds, distributed over six intensive care units. This took place in all six ICUs, all of which were included in the study.

The employment contracts of the professionals working at this institution includes different types. Employees can be hired on temporary contracts, according to the specific needs of the institution, or under the Consolidation of Labor Laws, through a foundation. In addition, the majority of professionals are tenured civil servants. The working day is organized into three shifts: morning and afternoon, each lasting 6 hours, and night, with a 12-hour period. In addition, employees in administrative positions work a regular 8-hour day.

### Period

Data were collected from May to November 2021.

### Population and sample

The population consisted of 202 nursing professionals. The calculation was carried out using the methodology for determining the estimate of a sample size for a proportion. The sample calculation process used a proportion p equal to 0.50, which represents the maximum variability of the binomial distribution, thus generating an estimate with the maximum practicable sample size. The sample corresponded to 133 professionals, and 114 subjects, who were selected by convenience, took part in the study.

### Selection criteria

The sample included professionals who provided direct care to patients - nursing technicians and clinical registered nurses - as well as nurses who performed management functions in ICUs. Professionals who, at the time of data collection, were on leave of absence or other types of legal leave were excluded.

### Variables and instruments

The variables age (in complete years), gender (female, male, prefer not to declare), position (nursing technician or registered nurse), length of professional experience (in years), work shift (day, night and others), work unit, working in more than one job (yes or no) and weekly working hours were collected using the personal and professional characterization form.

The Brazilian versions of the following instruments, previously validated for the target population, were used to collect data on the work environment, burnout and satisfaction: Practice Environment Scale (PES)^(16)^, the Maslach Burnout Inventory (MBI)^(17)^ and the job satisfaction subscale of the Safety Attitudes Questionnaire (SAQ) - Short form 2006^(18)^.

PES^(16)^ aims to assess professionals’ perceptions of the practice environment. The scale contains 24 items divided into five subscales: 1) nurse participation in hospital affairs; 2) nursing foundations for quality care; 3) nurse manager ability, leadership and support; 4) staffing and resource adequacy; 5) nurse-physician collegial relations. The answer options are presented using a 4-point Likert scale: 1 (strongly disagree), 2 (disagree), 3 (agree) and 4 (strongly agree). Scores of 2.5 or more on four or five subscales are classified as favorable environments; scores of 2.5 or more on two or three subscales are considered mixed environments and scores of more than 2.5 on none or only one subscale are evaluated as unfavorable environments for practice^(1)^. For this study, the reliability values, assessed by using Cronbach’s alpha, were: 0.80 for nurse participation in hospital affairs; 0.79 for nursing foundations for quality care; 0.85 for nurse manager ability, leadership and support; 0.71 for staffing and resource adequacy; and 0.76 for collegial nurse-physician relations.

The Maslach Burnout Inventory (MBI)^(17)^ was used to assess the burnout level among professionals. It consists of 22 items divided into three dimensions: emotional exhaustion, depersonalization and personal fulfillment, assessed on a Likert scale. The score for emotional exhaustion ranges from nine to 45 points, and for depersonalization from five to 25 points, with higher scores for both indicating a higher burnout level. The personal fulfillment dimension ranges from eight to 40 points and is inversely proportional to the burnout level, i.e. the lower the score, the higher the level of perceived burnout. The scores obtained in each dimension were divided into tertiles, classified as low, moderate or high. For this study, reliability, assessed using Cronbach’s alpha, was 0.89 for emotional exhaustion, 0.63 for depersonalization and 0.77 for decreased personal fulfillment. A license was purchased from MindGarden^©^ in order to use MBI.

The job satisfaction variable, derived from the job satisfaction subscale of the Safety Attitudes Questionnaire^(18)^ consists of five items assessed on a five-point Likert scale. Mean scores of 75 points or more were classified as job satisfaction^(18)^. For this study, the reliability of this subscale was 0.80, as measured by Cronbach’s alpha.

### Data collection

Data were collected using an online form. The link was sent to the corporate e-mail address only once by the nursing managers and made available via QRCode on posters in the nursing stations, with information about the target audience.

### Data processing and analysis

In order to analyze the data, a descriptive analysis was carried out on the qualitative variables using absolute and relative frequencies, while position and dispersion measures were used for the quantitative variables. For comparative analysis, unpaired Student’s t- or Mann-Whitney tests and Analysis of Variance (ANOVA) were used, followed by Tukey’s post-test, and Kruskal-Wallis, followed by Dunn’s post-test. Pearson’s or Spearman’s coefficients were used to analyze the correlations^(19)^. They were interpreted as weak (0.1 to 0.29), moderate (0.30 to 0.49) and strong (≥ 0.50)^(19)^. The definition of whether the comparison and correlation tests applied would be parametric or non-parametric was based on assessing the distribution of the data by constructing boxplots, assessing the presence of outliers and using the Shapiro-Wilk test. These analyses were carried out using the Statistical Analysis System (SAS), version 9.4, and the Statistical Package for the Social Sciences (SPSS), version 23^(20)^.

To investigate the presence of a mediating effect of job satisfaction in the relationship between the environment and burnout (dependent variable), structural equation modeling was used with the Partial Least Squares (PLS) method^(21)^ in the Smart PLS 3.3.5 software^(22)^. Complete mediation was considered when the indirect effect was significant, and the direct effect was not; partial mediation when both were significant; and no mediation when the indirect effect was not significant^(21)^.

### Ethical aspects

This study was approved by the Research Ethics Committee, according to Report no. 3.717.632. Participation was voluntary, following the acceptance of the Informed Consent Form, which was made available in digital format.

## Results

The sample consisted of 114 nursing professionals, namely 35.1% (n = 40) registered nurses and 64.9% (n = 74) nursing technicians. Of these, 78.1% (n = 89) were females, with a mean age of 35.5 years (Standard Deviation (SD) = 7.91). As for their place of work, 54.4% (n = 64) reported working in COVID ICUs and 45.6% (n = 52) in clinical and surgical ICUs.

As for professional experience, they reported an average of 7.27 years (SD = 5.85) in the job and an average of 3.73 years (SD = 4.58) in ICUs. Additionally, 46.49% (n = 53) had a second job; the average weekly workload was 48.14 hours. The majority of employees worked at night (45.6%, n = 52).

Table 1 shows the results of the subscales of the Practice Environment Scale and the job satisfaction subscale. It can be seen that the professionals reported a favorable perception of the environment, with means above 2.5, ranging from 2.64 to 3.09, and job satisfaction (M = 79.69).

**Table 1-:** Perception of nursing professionals according to the subscales of the Practice Environment Scale and job satisfaction*.* Ribeirão Preto, SP, Brazil, 2021

**Variables**	**Mean**	**Standard deviation**
Nurse participation in hospital affairs	2.64	0.73
Nursing foundations for quality care	2.83	0.59
Staffing and resource adequacy	3.01	0.61
Nurse manager ability, leadership and support	3.06	0.72
Collegial nurse-physician relations	3.09	0.60
Job satisfaction	79.69	17.52

In order to classify the burnout level, we considered the distribution of values in tertiles, low, medium and high, for each burnout dimension (Table 2). The level of emotional exhaustion was low (≤ 22; 37.7%); personal fulfillment was high (≤ 29; 38.6%) and depersonalization was moderate (> 7.63 and ≤ 11; 39.5%).

**Table 2-:** Burnout level reported by nursing professionals. Ribeirão Preto, SP, Brazil, 2021

**Burnout dimensions**	**Low**	n*	**%**	**Moderate**	n*	**%**	**High**	n*	**%**
Emotional exhaustion	≤ 22	43	37.7	> 22 e ≤ 27.3	33	29	> 27.3	38	33.3
Personal fulfillment	> 33	35	30.7	> 29 e ≤ 33	35	30.7	≤ 29	44	38.6
Depersonalization	≤ 7.63	38	33.3	> 7.63 e ≤ 11	45	39.5	> 11	31	27.1

*n = Sample

In the comparison analysis between the study variables, including personal and professional characteristics, perception of the practice environment, burnout and job satisfaction, those showing statistically significant differences were consolidated, such as emotional exhaustion, personal fulfillment, work shift, and staffing and resource adequacy (Table 3).

**Table 3-:** Comparison of nursing professionals’ perception of the MBI* dimensions and the PES^†^ subscales in relation to gender, function and shift. Ribeirão Preto, SP, Brazil, 2021

**Variables**			**Mean**	**Standard deviation**	**p-value**
MBI* - Emotional exhaustion	Gender	Male	22.58	7.77	0.0318 ^‡^
		Female	25.84	6.15	
MBI* - Personal fulfillment	Position	Technician	31.84	4.17	0.0078 ^‡^
		Nurse	29.63	4.14	
MBI* - Emotional exhaustion	Shift	Morning	28.07	6.78	0.0147 ^§||^
		Afternoon	20.88	6.27	
		Night	25.04	6.49	
		Administration	26.27	6.13	
PES ^†^ - Staffing and resource adequacy	Shift	Morning	2.89	0.82	0.0141 ^¶^ **
		Afternoon	3.45	0.47	
		Night	2.92	0.55	
		Administration	2.96	0.58	

*MBI = Maslach Burnout Inventory; ^†^PES = Practice Environment Scale; ^‡^p-value obtained using the unpaired Student’s t-test; ^§^p-value obtained using the ANOVA test;^||^Tukey’s post-test (Significant difference between morning and afternoon); ^¶^p-value obtained using the Kruskal-Wallis test; **Dunn’s post-test (Significant difference between afternoon and night)

Women showed higher levels of exhaustion as compared to men (p = 0.0318); professionals on the morning shift reported a higher level of emotional exhaustion than those on the afternoon and night shifts (p = 0.0147), and nursing technicians had a higher level of personal fulfillment than nurses (p = 0.0078). In addition, the perception of staffing and resource adequacy was higher on the afternoon shift as compared to the night shift (p = 0.0141).

Table 4 shows the correlations between the burnout dimensions, subscales of the practice environment and job satisfaction. There was a strong correlation between job satisfaction and the dimensions of emotional exhaustion (r = -0.54) and personal fulfillment (r = 0.58). Job satisfaction also showed strong correlations with the subscales Nursing foundations for quality care (r = 0.57), Nurse manager ability, leadership and support (r = 0.54) and Collegial nurse-physician relations (r = 0.55).

**Table 4-:** Correlation between MBI* dimensions, PES subscales^†^ and the SAQ^‡^ job satisfaction subscale. Ribeirão Preto, SP, Brazil, 2021

**Variables**	MBI* - Emotional exhaustion	MBI* - Personal fulfillment	MBI* - Depersonalization	PES ^†^ - Nurse participation in hospital affairs	PES ^†^ - Nursing foundations for quality care	PES ^†^ - Nurse manager ability, leadership and support	PES ^†^ - Staffing and resource adequacy	PES ^†^ - Collegial nurse-physician relations
PES ^†^ - Nurse participation in hospital affairs	-0.33 ^§||^	0.37 ^§||^	-0.15 ^||^					
PES ^†^ - Nursing foundations for quality care	-0.42 ^§¶^	0.46 ^§¶^	-0.24 ^§^					
PES ^†^ - Nurse manager ability, leadership and support	-0.46 ^§||^	0.42 ^§||^	-0.30 ^§||^					
PES ^†^ - Staffing and resource adequacy	-0.43 ^§||^	0.26 ^§||^	-0.13 ^||^					
PES ^†^ - Collegial nurse-physician relations	-0.47 ^§||^	0.47 ^§||^	-0.38 ^§||^					
SAQ ^‡^ - Job satisfaction	-0.54 ^§||^	0.58 ^§||^	-0.35 ^§||^	0.47 ^§||^	0.57 ^§||^	0.54 ^§||^	0.37 ^§||^	0.55 ^§||^

*MBI = Maslach Burnout Inventory; ^†^PES = Practice Environment Scale; ^‡^SAQ = Safety Attitudes Questionnaire; ^§^p-value < 0.05; ^||^Spearman’s correlation coefficient. Correlation strength (positive and negative values): Strong ≥ 0.50; moderate: 0.30 to 0.49; weak: 0.1 to 0.29; ^¶^Pearson correlation coefficient

**Figure 1-:**
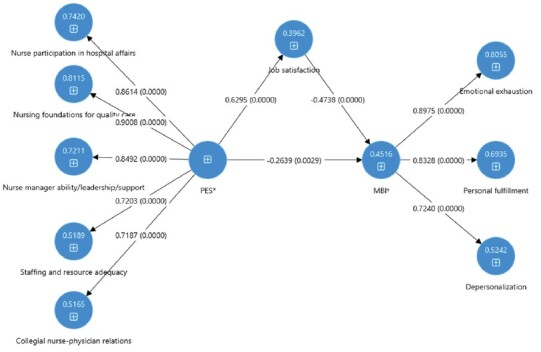
Theoretical model. Ribeirão Preto, SP, Brazil, 2021

Based on the literature review and the correlations found, a theoretical model was proposed, which is shown in Figure 1. The practice environment was defined as an independent variable; burnout as a dependent variable, and job satisfaction was classified as a mediating variable. The direct effect of the practice environment (PES) on burnout (MBI) showed a coefficient of -0.26, with a 95% confidence interval (CI) ranging from -0.44 to -0.08. The indirect effect, mediated by job satisfaction, had a coefficient of -0.30, with a 95% CI ranging from -0.41 to -0.19. The adjusted model showed an R² value of 45.2% for the MBI variable. Both the direct and indirect effects were statistically significant.

*PES = Practice Environment Scale; ^†^MBI = Maslach Burnout Inventory

## Discussion

This study aimed to analyze, in the light of a theoretical model, the relationship between the perception of nursing professionals in intensive care units about the practice environment, burnout levels and job satisfaction.

The data collection period coincided with the second wave of the COVID-19 pandemic in Brazil. At that time, the country was experiencing significant fluctuations in the numbers of cases; deaths were significant; vaccination of health professionals was in its early stages and the spread of the virus variants raised additional concerns about the transmissibility and efficacy of vaccines^(23)^. It is important to note that this context and the working conditions of that period may have influenced professionals’ perceptions of the work environment, burnout levels and job satisfaction.

The professionals in this study were predominantly female, a characteristic attributed to a historical construction of the professional nursing category^(24)^. The average age was 35.5 years, and it included an average time of experience in the job of 7.27 years. Although the working hours at the institution are 30 hours a week for civil servants and 36 hours for private contracts, the subjects reported an average weekly workload of 48.14 hours, which may be associated with a second job, as almost half of the nursing professionals were involved in more than one job. This situation may be a response to the need to balance financial demands and the search for professional stability. Dual employment can present challenges in terms of burnout, personal time availability and general health, negatively affecting both the physical and emotional balance of professionals^(25)^.

The distribution of nursing professionals in intensive care units represented the need of the period, in terms of redirecting and hiring staff dedicated to the intensive treatment of COVID-19. The change required nurses to quickly adapt and update their skills, as caring for patients presented specific challenges and additional protection requirements due to the contagious nature of the virus^(1,25)^. Although the practice environment analyzed had undergone such adjustments generated by the pandemic, nursing professionals reported a favorable perception of such practice environment, as in other studies^(14,26)^.

As for the classification of the burnout level, the majority of professionals showed low emotional exhaustion, high personal fulfillment and moderate depersonalization. Taken together, the MBI results indicate a complex picture. Although the majority of professionals reported low emotional exhaustion, the proportion of participants with moderate to high emotional exhaustion cannot be disregarded, which suggests that the health crisis may have intensified the emotional demands on professionals, possibly due to the increased workload, exposure to more stressful situations and facing unknown challenges^(3,27-28)^.

In addition, the level of depersonalization reported can be attributed to adverse situations, such as the need for distancing and safety protocols, which may have led to an emotional detachment between professionals and patients. On the other hand, the professionals who reported a feeling of personal fulfillment may be indicative of the resilience and sense of purpose that they found when dealing with the crisis, due to the recognition of the importance of their work in the pandemic scenario^(29)^.

With regard to job satisfaction, the result reflects that individuals are satisfied in relation to their work activities, i.e. they have a perception that their expectations, needs and objectives are met in the context of work. A study evaluating the relationship between perception of the nursing practice environment and job satisfaction among intensive care nurses found that a favorable practice environment significantly correlated with job satisfaction, further highlighting the importance of a healthy environment for positive nursing outcomes^(14)^.

When comparing emotional exhaustion between men and women, women have a higher level of exhaustion than men. Some possible causes to consider include the existence of a sizable segment of professionals who have more than one job coupled with domestic activities. This combined overload can significantly increase the levels of stress and emotional exhaustion among female professionals^(25)^.

There was a lower level of personal fulfillment among nurses. They usually carry out administrative and staff management duties, make clinical decisions and provide less direct care to patients, situations which can affect the perception of personal fulfillment. Also among nurses, there may be greater expectations regarding the leadership role and relationships with coordination. Such responsibilities can be an extra source of stress and pressure, affecting the perception of personal fulfillment^(25,29)^.

The level of emotional exhaustion between shifts also showed a significant difference. The higher score on the morning shift indicates that nursing professionals have a higher level of emotional exhaustion than those on the other shifts. The morning shift generally has a higher demand for test collection, referrals for surgery and multi-professional consultations. This shift can involve a series of tasks and procedures that need to be carried out at the start of the day. These intense and concentrated demands can overload nursing professionals and contribute to feelings of emotional exhaustion^(27)^.

With regard to staffing and resource adequacy, night-shift professionals scored the lowest and differed from afternoon-shift professionals. It is known that during the night, access to support services, such as maintenance and IT, and contact with hospital managers is limited, as they work administrative hours. These aspects can contribute to a perception of insufficient resources on night shifts. Health care professionals operating in environments with limited or inadequate resources often face a heavy workload, which increases the risk of generating emotional exhaustion, reduced productivity and professional dissatisfaction. These problems lead to an increased risk of errors and reduce the quality of the care provided^(2,30)^.

In the analysis of correlations, a strong inverse correlation was observed between emotional exhaustion and job satisfaction, which can be explained by the nature of the work environment, i.e. intense demands, emotional burden and excessive stress favor a decrease in job satisfaction and an increase in emotional exhaustion, affecting the well-being and motivation of professionals. Studies have shown that better working environments have been correlated with lower rates of job dissatisfaction and lower burnout levels^(14,16)^.

Furthermore, the strong positive correlation between job satisfaction, personal fulfillment and the PES variables can be interpreted as follows: job satisfaction is linked to the feeling of personal fulfillment and the perception that work is in line with individual goals and values. Satisfied professionals tend to be more engaged and motivated to provide higher quality care to patients. A work environment where leaders offer support and promote a positive climate can foster professional satisfaction and performance. Positive and collaborative relationships between nurses and physicians can improve communication, coordination and team effectiveness^(31)^.

On the other hand, there were also moderate correlations between emotional exhaustion and all the PES domains: professionals who feel they do not have the opportunity to influence decisions about their work and the environment around them can experience feelings of emotional exhaustion. Lack of autonomy can make professionals feel devalued and demotivated^(32)^. In addition, if professionals do not feel that they are receiving adequate support from the organization, that can increase the level of emotional exhaustion. Lack of communication, lack of emotional support and lack of clarity about the institution’s policies all contribute to this scenario^(31)^. When the resources needed to perform one’s job properly are scarce, professionals can also feel exhausted^(11)^.

Another important aspect is the moderate inverse correlation between job satisfaction and depersonalization. This shows that when nursing technicians and nurses are satisfied with their work, there is a tendency for depersonalization to be lower. Satisfied professionals generally have greater empathy and connection with patients, which can help avoid depersonalization, a phenomenon in which professionals treat patients in an impersonal way^(14)^.

Regarding the moderate positive correlation between nurse participation in hospital affairs and the availability of adequate resources, when the nursing staff feel valued and listened to in relation to hospital issues, their satisfaction increases. This may be associated with a greater sense of importance and influence in the workplace. This feeling of being valued is reinforced when the nursing staff perceive that they have sufficient resources at their disposal, resulting in greater job satisfaction^(9,11)^.

The correlations identified in this study, in alignment with the proposed theoretical model, confirmed the relationship between the practice environment variables and burnout. This relationship remains present regardless of the inclusion of the job satisfaction variable in the model. The finding of partial mediation means that the relationship between practice environment and burnout is explained both directly and indirectly by job satisfaction, i.e. there are other factors besides satisfaction that can also mediate the way in which the work environment affects burnout. A favorable practice environment, with a lower burnout level, encompasses the availability of adequate resources, effective support systems, fluid communication between teams and continuous learning opportunities, which in turn enables the application of practices in scientific studies, stimulates interdisciplinary collaboration and fosters safe patient care^(14,30)^.

In the opposite direction, working with inadequate or obsolete equipment and a lack of essential resources can increase the pressure on health professionals, leading to stress, emotional exhaustion and an insensitive attitude towards work and patients. This can lead to inadequate health care and affect patient safety^(5,30)^.

In the COVID-19 pandemic, there were numerous reports and observations of high workloads in hospitals. This state of exhaustion and the strenuous demands put health care workers at significant risk of developing physical and mental exhaustion. Many of them faced extremely complicated situations, dealing with an increasing number of patients, a lack of adequate resources and the constant threat of infection. For this reason, the discussion on workload is closely linked to assessing the sufficiency and effectiveness of human resources within a nursing practice environment^(2)^.

Another point for discussion in this relationship is the lack of support from the administration and managers, which received the lowest score among the PES subscales evaluated in this study. Lack of support can contribute to burnout, as it can make professionals feel undervalued. The lack of feedback, recognition and opportunities for growth can lead to a feeling of stagnation, contributing to low personal fulfillment, a negative work culture and increased turnover^(11)^.

With regard to feelings of depersonalization and low personal fulfillment, they can also be associated with the relationship between nurses and physicians. In a hostile working environment, where there is rivalry and a lack of respect, conflicting attitudes and a disconnection with work and colleagues are expected. This is detrimental to team collaboration, treatment effectiveness and patient satisfaction, meaning that collegial relationships between nurses and physicians are fundamental to ensuring a safe and collaborative health care environment. This exchange of knowledge can lead to better treatment decisions and attention to patients’ needs^(2,27,30)^.

In general, the practice environment can have a direct and profound effect on health professionals’ burnout. Understanding these relationships is key to creating effective strategies to prevent and treat burnout. Interventions can include improving communication within the team, providing adequate resources, adjusting the workload and promoting a healthy organizational culture.

Among the limitations of this study was the difficulty in collecting data in the midst of the health crisis. The data were collected remotely, and it was not possible to monitor and control the number of beds and professionals, which fluctuated due to the pandemic waves.

## Conclusion

Nursing professionals reported a favorable perception of the practice environment in all PES subscales. As regards burnout, most reported low emotional exhaustion, high personal fulfillment and moderate depersonalization, as well as being satisfied with their work. The study showed, by using the theoretical model, that actions to improve the practice environment can reduce the burnout levels, both directly and by increasing job satisfaction.

However, job satisfaction does not fully explain this relationship, suggesting that there are other factors not examined in this model that can also influence burnout. The results highlight the complexity of burnout, implying that even in workplaces that have a favorable and satisfying practice environment, some employees still face difficulties related to it.

In practical terms, strategies such as emotional support, open communication, continuous learning and monitoring of these variables in the post-pandemic period are recommended, with the aim of mitigating the negative impacts of the pandemic and favoring a healthier and more balanced work environment.

The results of this study have relevant implications for professional nursing practice, especially in the context of intensive care units. The identification of job satisfaction as a partial mediator between the practice environment and burnout highlights the importance of institutional strategies aimed at improving the organizational environment, such as valuing professionals, strengthening leadership, improving working conditions and promoting collaborative relationships between teams. Such actions can contribute directly to reducing burnout and promoting the occupational health of professionals. The study also proposes a theoretical model that broadens the understanding of the interactions between organizational and psychosocial variables in the context of nursing and may support further research. It is recommended that future studies explore other potential mediators and moderators of this relationship, including individual, organizational and cultural aspects, as well as longitudinal research that allows the variables to be monitored over time and in different care-provision settings.

## Data Availability

Datasets related to this article will be available upon request to the corresponding author.
