# Supplementary material for: Practice environment and burnout in intensive care units: is job satisfaction a mediator in this relationship?
Source: Rev Lat Am Enfermagem. 2025 Nov 3;33:e4713. doi: 10.1590/1518-8345.7659.4713 (PMC12587757; doi:10.1590/1518-8345.7659.4713)
Supplement: Translation

# Translation: Ambiente de prática e *burnout* em unidades de terapia intensiva: a satisfação no trabalho é uma mediadora dessa relação?*

**DOI:** 10.1590/1518-8345.7659.4714

**Authors:** Jussara Aparecida da Silva Furlan, Edinêis de Brito Guirardello

**Affiliations:** 1 Universidade Estadual de Campinas, Faculdade de Enfermagem, Campinas, SP, Brasil

**Keywords:** Enfermagem, Esgotamento Profissional, Ambiente de Instituições de Saúde, Satisfação no Emprego, Unidades de Terapia Intensiva, Exaustão Emocional

## Abstract

analisar, com base em um modelo teórico, a relação entre a percepção dos profissionais de enfermagem em unidades de terapia intensiva sobre o ambiente de prática, o nível de *burnout* e a satisfação no trabalho.

estudo transversal e correlacional, realizado com 114 profissionais de enfermagem de um hospital de ensino do noroeste paulista. Utilizaram-se a ficha de caracterização pessoal e profissional, a *Practice Environment Scale,* o Inventário de *Burnout* Maslach e a subescala satisfação no trabalho do *Safety Attitudes Questionnaire*. Foram realizadas análises descritivas, testes de comparação (t de Student não pareado, Mann-Whitney, ANOVA e Kruskal-Wallis), correlações (Pearson ou Spearman) e modelagem de equações estruturais para verificar o efeito mediador.

os profissionais reportaram um ambiente favorável à prática, satisfação no trabalho, baixo nível de exaustão emocional, elevado nível de realização pessoal e moderado nível de despersonalização. Verificou-se que a satisfação no trabalho resultou em efeito mediador parcial entre ambiente de prática e o *burnout*.

ações voltadas à melhoria do ambiente de prática podem reduzir diretamente o *burnout* ou mediá-lo por meio do aumento da satisfação no trabalho. Compreender essas relações é fundamental para promover um ambiente favorável, minimizar o *burnout* e ampliar a satisfação no trabalho.

## Destaques

**(1)** O estudo confirma a relação entre o ambiente de prática e o *burnout*. **(2)** A satisfação no trabalho medeia parcialmente a relação entre o ambiente e o *burnout*. **(3)** Melhorar o ambiente reduz o *burnout*, tanto de forma direta, quanto pelo aumento da satisfação. **(4)** O modelo teórico auxilia gestores a identificarem intervenções específicas.

## Introdução

O ambiente de prática da enfermagem é compreendido pelas características organizacionais do ambiente de trabalho que a facilitam ou dificultam^(1)^. Em Unidades de Terapia Intensiva (UTIs), esse ambiente é altamente especializado e multidimensional, tendo como finalidade principal fornecer cuidado intensivo a pacientes gravemente enfermos. Os profissionais de enfermagem que trabalham nessas unidades desempenham atividades voltadas à estabilização e recuperação dos pacientes, estando também sujeitos a impactos emocionais decorrentes das exigências e características desse local de trabalho^(2-3)^.

Nos últimos anos, as atividades realizadas pela enfermagem ganharam destaque devido ao seu papel nos cuidados a pacientes com COVID-19. As UTIs foram os ambientes com maior demanda por atendimento à saúde, passando por um processo de expansão do número de leitos e aumento da necessidade de recursos humanos e materiais. Esse cenário de prática impôs aos profissionais de enfermagem a realização de suas atividades diante da escassez de recursos e a sobrecarga de trabalho^(2)^, além de presenciarem inúmeras mortes, o adoecimentos de colegas, o próprio adoecimento e o medo constante de levar o vírus para casa^(4)^, caracterizando um ambiente de prática propício ao adoecimento profissional^(3,5)^.

Diante desse contexto de sobrecarga e adversidades enfrentadas durante a pandemia, é fundamental compreender quais elementos do ambiente de trabalho contribuem para a saúde e o desempenho da equipe de enfermagem. Considera-se o ambiente de trabalho favorável quando ele possibilita que a equipe de enfermagem exerça suas atividades com autonomia, dispõe de recursos humanos e materiais adequados, conta com gestores que mantêm uma comunicação aberta e oferecem oportunidades de desenvolvimento profissional. Em contrapartida, um ambiente desfavorável é caracterizado pela escassez de suprimentos, sobrecarga de trabalho, ausência de apoio da liderança, falta de colaboração entre as equipes de saúde, insatisfação e *burnout* entre os profissionais^(6-7)^.

Condições de saúde diretamente relacionadas ao ambiente de trabalho são amplamente descritas na literatura, dentre elas o *burnout*, um problema frequente entre profissionais de enfermagem. Essa síndrome está associada à pressão excessiva no ambiente de trabalho, resultando em exaustão emocional, despersonalização e redução da realização pessoal^(8)^. Estudos apontam altas taxas de *burnout* entre enfermeiros que atuam em UTIs^(3,8)^, com impactos negativos tanto na segurança do paciente^(9)^ quanto na qualidade do atendimento^(10-11)^.

Uma revisão sistemática analisou 85 estudos envolvendo 288.581 enfermeiros de 32 países e identificou uma prevalência média de *burnout* de 30,7% (±9,7%). O *burnout* foi associado a desfechos negativos, como pior clima de segurança, maior incidência de quedas de pacientes, erros de medicação, cuidados omitidos, menor qualidade do cuidado e insatisfação dos pacientes, independentemente da idade, sexo, experiência profissional ou localização geográfica^(9)^.

A relação entre *burnout* e satisfação no trabalho tem sido estudada entre profissionais de enfermagem que atuam em unidades de terapia intensiva. O *burnout* e a satisfação no trabalho apresentam uma relação inversamente proporcional entre si. Um estudo de revisão sistemática recente, envolvendo 18 estudos e um total de 3.168 enfermeiros de UTI, identificou uma prevalência geral de *burnout* de 37,5% e revelou uma correlação inversa significante entre *burnout* e satisfação no trabalho, evidenciando que profissionais mais satisfeitos apresentam menor risco de desenvolver a síndrome^(12)^. Altos níveis de exaustão emocional e despersonalização estão associados à diminuição da satisfação profissional, impactando diretamente a motivação, o comprometimento e a qualidade da assistência prestada. Por outro lado, profissionais satisfeitos tendem a apresentar menor nível de *burnout*, maior resiliência frente às adversidades do ambiente e melhor percepção do próprio desempenho^(13)^.

Nesse contexto, a satisfação no trabalho pode atuar como fator protetor frente ao esgotamento emocional e ao desgaste profissional. A mesma metanálise apontou que profissionais satisfeitos demonstram maior resiliência, melhor desempenho e menores índices de absenteísmo e rotatividade^(12)^. A satisfação da equipe de enfermagem não se refere apenas ao bem-estar individual de seus membros e à percepção sobre a vida profissional, mas também à qualidade e à saúde do ambiente de trabalho. A gestão ineficiente do ambiente de prática, associada a altos níveis de exaustão emocional, tem sido relacionada ao aumento da insatisfação no trabalho entre os profissionais de enfermagem^(9)^.

Considerando essa relação, quando analisados em conjunto o ambiente de prática, o *burnout* e a satisfação no trabalho, verifica-se que ambientes positivos estão correlacionados a menores níveis de *burnout* e menores níveis de insatisfação profissional^(7,14)^. A análise integrada desses aspectos permite compreender a contribuição do ambiente de prática para os resultados da enfermagem e os desfechos para os pacientes, possibilitando aos gestores identificar pontos que necessitam de melhorias^(1)^. Isto significa que um ambiente considerado favorável à prática de enfermagem pode contribuir com repercussões positivas para pacientes, profissionais e organizações^(15)^.

Apesar do volume crescente de pesquisas sobre o ambiente de prática da enfermagem, ainda são escassos os estudos que investigam, de forma integrada, a relação entre ambiente de prática, *burnout* e a satisfação no trabalho dos profissionais de enfermagem. A literatura apresenta avanços na compreensão individual desses elementos, porém, há uma lacuna quanto à análise conjunta desses fatores e à proposição de modelos teóricos que expliquem suas interações.

Diante da relevância do ambiente de prática no esgotamento profissional e na satisfação dos profissionais de enfermagem em UTIs, este estudo parte da seguinte questão norteadora: a satisfação no trabalho medeia a relação entre o ambiente de prática e o *burnout* em profissionais de enfermagem que atuam em UTIs? Portanto, o objetivo deste estudo foi analisar, com base em um modelo teórico, a relação entre a percepção dos profissionais de enfermagem em unidades de terapia intensiva sobre o ambiente de prática, o nível de *burnout* e a satisfação no trabalho.

## Método

### Tipo

Estudo transversal e correlacional, que seguiu as recomendações do *Strengthening the Reporting of Observational Studies in Epidemiology* (STROBE).

### Local

Estudo realizado em unidades de terapia intensiva para adultos em um hospital de ensino na cidade de Ribeirão Preto, SP, Brasil. Esse hospital foi referência para os casos críticos de COVID-19 de sua área de abrangência e, por esse motivo, passou por expansão do número de leitos intensivos. No início da coleta de dados, detinha 84 leitos intensivos, distribuídos em seis Unidades de Terapia Intensiva. Esta ocorreu nas seis UTIs, as quais foram todas incluídas no estudo.

Os vínculos dos profissionais que atuam nessa instituição abrangem diferentes modalidades de contratação. Os colaboradores podem ser contratados por meio de contratos temporários, seguindo as necessidades específicas da instituição, ou pelo regime da Consolidação das Leis do Trabalho, por meio de uma Fundação. Além disso, a maioria dos profissionais é composta por concursados. A jornada de trabalho é organizada em três turnos: manhã e tarde, com duração de 6 horas cada, e noite, com um período de 12 horas. Adicionalmente, os colaboradores em funções administrativas cumprem um expediente regular de 8 horas diárias.

### Período

A coleta de dados foi realizada entre maio e novembro de 2021.

### População e amostra

A população foi composta por 202 profissionais de enfermagem. O cálculo foi realizado utilizando a metodologia para determinar a estimativa de um tamanho amostral para uma proporção. No processo do cálculo amostral utilizou-se uma proporção p igual a 0,50, que representa a variabilidade máxima da distribuição binomial, gerando, desse modo, uma estimativa com o tamanho amostral máximo praticável. A amostra correspondeu a 133 profissionais, e participaram do estudo 114 sujeitos, selecionados por conveniência.

### Critérios de seleção

Foram considerados na amostra os profissionais que atuavam na assistência direta aos pacientes – técnicos de enfermagem e enfermeiros assistenciais – bem como os enfermeiros que exerciam funções de gestão nas UTIs. Foram excluídos os profissionais que, no momento da coleta de dados, encontravam-se afastados por motivo de licença ou outros tipos de afastamento legal.

### Variáveis e instrumentos

As variáveis idade (em anos completos), sexo (feminino, masculino, prefiro não declarar), função (técnico de enfermagem ou enfermeiro), tempo de experiência profissional (em anos), turno de trabalho (diurno, noturno e outros), unidade de trabalho, presença de mais de um vínculo empregatício (sim ou não) e carga horária semanal (em horas) foram coletadas por meio da ficha de caracterização pessoal e profissional.

Para a coleta de dados relacionadas ao ambiente de trabalho, *burnout* e satisfação, foram utilizadas as versões brasileiras dos seguintes instrumentos, previamente validados para a população-alvo: *Practice Environment Scale* (PES)^(16)^, Inventário de *Burnout* Maslach (IBM)^(17)^ e a subescala satisfação no trabalho do *Safety Attitudes Questionnaire* (SAQ) – *Short form 2006* ^(18)^.

A PES^(16)^ tem por finalidade avaliar a percepção dos profissionais sobre o ambiente da prática. A escala contém 24 itens distribuídos em cinco subescalas: 1) participação dos enfermeiros na discussão dos assuntos hospitalares 2) fundamentos de enfermagem voltados para a qualidade do cuidado; 3) habilidade, liderança e suporte dos coordenadores/supervisores de enfermagem aos enfermeiros/equipe de enfermagem; 4) adequação da equipe e de recursos; 5) relações colegiais entre profissionais de enfermagem e médicos. As opções de respostas são apresentadas por meio de uma escala Likert de 4 pontos: 1 (discordo totalmente), 2 (discordo), 3 (concordo) e 4 (concordo totalmente). Pontuações iguais ou superiores a 2,5 em quatro ou cinco subescalas são classificadas como ambientes favoráveis; pontuações iguais ou superiores a 2,5 em duas ou três subescalas são consideradas ambientes mistos e resultados superiores a 2,5 pontos, em nenhuma ou apenas em uma subescala, são avaliados como ambientes desfavoráveis à prática^(1)^. Para o presente estudo, os valores de confiabilidade, avaliados por meio do alfa de Cronbach, foram de 0,80 para participação dos enfermeiros na discussão dos assuntos hospitalares; 0,79 para fundamentos de enfermagem voltados à qualidade do cuidado; 0,85 para habilidade, liderança e suporte dos coordenadores/supervisores de enfermagem aos enfermeiros/equipe de enfermagem; 0,71 para adequação da equipe e de recursos e 0,76 para relações colegiais entre profissionais de enfermagem e médicos.

O Inventário de *Burnout* Maslach^(17)^ foi empregado para avaliar o nível de *burnout* dos profissionais. É composto por 22 itens distribuídos em três dimensões: exaustão emocional, despersonalização e realização pessoal, avaliadas em uma escala Likert. A pontuação da exaustão emocional varia de nove a 45 pontos, e a da despersonalização, de cinco e 25 pontos, sendo que, para ambas, pontuações mais altas indicam maior nível de *burnout*. A dimensão realização pessoal varia de oito a 40 pontos, sendo inversamente proporcional ao nível de *burnout*, ou seja, quanto menor a pontuação maior o nível de *burnout* percebido. Os escores obtidos em cada dimensão foram divididos em tercis, classificados como baixo, moderado ou alto. Para este estudo, a confiabilidade, avaliada por meio do alfa de Cronbach, foi de 0,89 para exaustão emocional, 0,63 para despersonalização e 0,77 para diminuição da realização pessoal. Para a aplicação do IBM, foi adquirida a licença junto à *MindGarden* ^©^.

A variável satisfação no trabalho, derivada da subescala satisfação no trabalho do *Safety Attitudes Questionnaire* ^(18)^, foi composta por cinco itens avaliados em uma escala Likert de cinco pontos. Pontuações médias iguais ou superiores a 75 pontos foram classificadas como satisfação no trabalho^(18)^. Para este estudo, a confiabilidade dessa subescala, medida pelo alfa de Cronbach, foi de 0,80.

### Coleta de dados

Os dados foram coletados por meio de um formulário *online*. O *link* foi enviado pelos gestores de enfermagem, uma única vez, para o *e-mail* corporativo e disponibilizado via *QRCode* por meio de cartazes nos postos de enfermagem, com a informação sobre o público-alvo.

### Tratamento e análise dos dados

Para a análise dos dados, realizou-se análise descritiva das variáveis qualitativas por meio de frequências absolutas e relativas, enquanto para as variáveis quantitativas foram utilizadas medidas de posição e dispersão. Na análise comparativa, foram utilizados os testes t de Student não pareado ou Mann-Whitney e *Analysis of Variance* (ANOVA), seguidos do pós-teste de Tukey, e Kruskal-Wallis, seguido do pós-teste de Dunn. Para as análises das correlações, foram utilizados os coeficientes de Pearson ou Spearman^(19)^_._ Elas foram interpretadas como fracas (0,1 a 0,29), moderadas (0,30 a 0,49) e fortes (≥ 0,50)^(19)^. A definição de se os testes de comparação e correlação aplicados seriam paramétricos ou não-paramétricos foi baseada na avaliação da distribuição dos dados por meio da construção de *boxplots*, com avaliação da presença de *outliers*, e por meio do teste de Shapiro-Wilk. Essas análises foram realizadas com o *Statistical Analysis System* (SAS), versão 9.4, e o *Statistical Package for the Social Sciences* (SPSS), versão 23^(20)^.

Para investigar a presença de efeito mediador da satisfação no trabalho na relação entre o ambiente e o *burnout* (variável dependente), utilizou-se a modelagem de equações estruturais com o método *Partial Least Squares* (PLS)^(21)^, no *software Smart* PLS 3.3.5^(22)^. Considerou-se mediação completa quando o efeito indireto foi significante e o direto, não; mediação parcial quando ambos foram significantes; e sem mediação quando o efeito indireto não foi significante^(21)^.

### Aspectos éticos

Este estudo foi aprovado pelo Comitê de Ética em Pesquisa, sob o Parecer nº. 3.717.632.

A participação foi voluntária, após o aceite do Termo de Consentimento Livre e Esclarecido, disponibilizado em formato digital.

## Resultados

A amostra foi constituída por 114 profissionais de enfermagem, sendo 35,1% (n = 40) enfermeiros e 64,9% (n = 74) técnicos de enfermagem. Desses, 78,1% (n = 89) eram do sexo feminino, com média de idade de 35,5 anos (Desvio-Padrão (DP) = 7,91). Quanto ao local de trabalho, 54,4% (n = 64) reportaram trabalhar nas UTIs COVID e 45,6% (n = 52) nas UTIs clínica e cirúrgica.

Quanto à experiência profissional, reportaram uma média de 7,27 anos (DP = 5,85) na função e uma média de 3,73 anos (DP = 4,58) no setor. Além disso, 46,49% (n = 53) possuíam outro vínculo empregatício; a carga horária semanal média foi de 48,14 horas. A maioria dos funcionários trabalhava no período noturno (45,6%, n = 52).

Na Tabela 1 estão apresentados os resultados das subescalas da *Practice Environment Scale* e da subescala satisfação no trabalho. Observa-se que os profissionais reportaram uma percepção favorável do ambiente, com médias superiores a 2,5 que variaram entre 2,64 e 3,09, e satisfação com o trabalho (M = 79,69).

**Tabela 1-:** Percepção dos profissionais de enfermagem segundo as subescalas da *Practice Environment Scale* e satisfação no trabalho*.* Ribeirão Preto, SP, Brasil, 2021

**Variáveis**	**Média**	**Desvio-padrão**
Participação dos enfermeiros na discussão dos assuntos hospitalares	2,64	0,73
Fundamentos de enfermagem voltados para a qualidade do cuidado	2,83	0,59
Adequação da equipe e de recursos	3,01	0,61
Habilidade, liderança e suporte dos coordenadores/supervisores de enfermagem aos enfermeiros/equipe de enfermagem	3,06	0,72
Relações colegiais entre profissionais de enfermagem e médicos	3,09	0,60
Satisfação no trabalho	79,69	17,52

Para a classificação do nível de *burnout*, foi considerada a distribuição dos valores em tercis, de nível baixo, médio e alto, para cada dimensão do *burnout* (Tabela 2). Destaca-se que o nível de exaustão emocional foi baixo (≤ 22; 37,7%); o de realização pessoal foi elevado (≤ 29; 38,6%) e o de despersonalização foi moderado (> 7,63 e ≤ 11; 39,5%).

**Tabela 2-:** Nível de *burnout* reportado pelos profissionais de enfermagem. Ribeirão Preto, SP, Brasil, 2021

**Dimensões do *burnout***	**Baixo**	n*	**%**	**Moderado**	n*	**%**	**Elevado**	n*	**%**
Exaustão emocional	≤ 22	43	37,7	> 22 e ≤ 27,3	33	29	> 27,3	38	33,3
Realização pessoal	> 33	35	30,7	> 29 e ≤ 33	35	30,7	≤ 29	44	38,6
Despersonalização	≤ 7,63	38	33,3	> 7,63 e ≤ 11	45	39,5	> 11	31	27,1

*n = Amostra

Na análise de comparação entre as variáveis do estudo, entre elas as características pessoais e profissionais, a percepção do ambiente de prática, o *burnout* e a satisfação no trabalho, foram consolidadas as que demonstraram diferenças estatisticamente significantes, como exaustão emocional, realização pessoal, turno de trabalho e adequação da equipe e de recursos (Tabela 3).

**Tabela 3-:** Comparação da percepção dos profissionais de enfermagem quanto às dimensões do IBM* e as subescalas da PES^†^ em relação ao sexo, função e turno. Ribeirão Preto, SP, Brasil, 2021

**Variáveis**			**Média**	**Desvio-padrão**	**p-valor**
IBM* - Exaustão Emocional	Sexo	Masculino	22,58	7,77	0,0318 ^‡^
		Feminino	25,84	6,15	
IBM* - Realização Pessoal	Função	Técnico	31,84	4,17	0,0078 ^‡^
		Enfermeiro	29,63	4,14	
IBM* - Exaustão Emocional	Turno	Manhã	28,07	6,78	0,0147 ^§||^
		Tarde	20,88	6,27	
		Noite	25,04	6,49	
		Administrativo	26,27	6,13	
PES ^†^ - Adequação da equipe e de recursos	Turno	Manhã	2,89	0,82	0,0141 ^¶^ **
		Tarde	3,45	0,47	
		Noite	2,92	0,55	
		Administrativo	2,96	0,58	

*IBM = Inventário de *Burnout* de Maslach; ^†^PES = *Practice Environment Scale*; ^‡^p-valor obtido por meio do teste t de Student não pareado; ^§^p-valor obtido por meio do teste Anova; ^||^Pós-teste de Tukey (Diferença significante entre Manhã e Tarde); ^¶^p-valor obtido por meio do teste de Kruskal-Wallis; **Pós-teste de Dunn (Diferença significante Tarde x Noite)

As mulheres apresentaram maiores níveis de exaustão em comparação aos homens (p = 0,0318); os profissionais do turno manhã reportaram maior nível de exaustão emocional em relação aos dos turnos tarde e noite (p = 0,0147), e os técnicos de enfermagem apresentaram maior nível de realização pessoal em comparação aos enfermeiros (p = 0,0078). Além disso, a percepção de adequação de equipe e recursos foi maior no turno da tarde em comparação ao noturno (p = 0,0141).

Na Tabela 4 estão apresentadas as correlações entre as dimensões do *burnout*, subescalas do ambiente de prática e a satisfação no trabalho. Verifica-se a existência de correlação de forte magnitude entre satisfação no trabalho e as dimensões exaustão emocional (r = -0,54) e realização pessoal (r = 0,58) e, correlação de forte magnitude entre satisfação no trabalho e as subescalas Fundamentos de enfermagem voltados para a qualidade do cuidado (r = 0,57), Habilidade, liderança e suporte dos gestores de enfermagem aos enfermeiros/equipe de enfermagem (r = 0,54) e Relações colegiais entre profissionais de enfermagem e médicos (r = 0,55).

**Tabela 4-:** Correlação entre as dimensões do IBM*, subescalas da PES^†^ e a subescala satisfação no trabalho do SAQ^‡^. Ribeirão Preto, SP, Brasil, 2021

**Variáveis**	IBM*- Exaustão Emocional	IBM*- Realização Pessoal	IBM* - Despersonalização	PES ^†^ - Participação dos enfermeiros na discussão dos assuntos hospitalares	PES ^†^ - Fundamentos de enfermagem voltados para a qualidade do cuidado	PES ^†^ - Habilidade, liderança e suporte dos gestores de enfermagem aos enfermeiros/equipe de enfermagem	PES ^†^ - Adequação da equipe e de recursos	PES ^†^ - Relações colegiais entre profissionais de enfermagem e médicos
PES ^†^ - Participação dos enfermeiros na discussão dos assuntos hospitalares	-0,33 ^§||^	0,37 ^§||^	-0,15 ^||^					
PES ^†^ - Fundamentos de enfermagem voltados para a qualidade do cuidado	-0,42 ^§¶^	0,46 ^§¶^	-0,24 ^§^					
PES ^†^ - Habilidade, liderança e suporte dos coordenadores/supervisores de enfermagem aos enfermeiros/equipe de enfermagem	-0,46 ^§||^	0,42 ^§||^	-0,30 ^§||^					
PES ^†^ - Adequação da equipe e de recursos	-0,43 ^§||^	0,26 ^§||^	-0,13 ^||^					
PES ^†^ - Relações colegiais entre profissionais de enfermagem e médicos	-0,47 ^§||^	0,47 ^§||^	-0,38 ^§||^					
SAQ ^‡^ - Satisfação no trabalho	-0,54 ^§||^	0,58 ^§||^	-0,35 ^§||^	0,47 ^§||^	0,57 ^§||^	0,54 ^§||^	0,37 ^§||^	0,55 ^§||^

*IBM = Inventário de *Burnout* Maslach; ^†^PES = *Practice Environment Scale*; ^‡^SAQ = *Safety Attitudes Questionnaire*; ^§^p-valor < 0,05; ^||^Coeficiente de correlação de Spearman. Intensidade da correlação (valores positivos e negativos): Forte ≥ 0,50; moderada: 0,30 a 0,49; fraca: 0,1 a 0,29; ^¶^Coeficiente de correlação de Pearson

A partir da revisão da literatura e das correlações encontradas, um modelo teórico foi proposto, o qual é apresentado na Figura 1. O ambiente de prática foi definido como uma variável independente; o *burnout* como uma variável dependente, e a satisfação no trabalho foi classificada como uma variável mediadora. O efeito direto do ambiente de prática (PES) sobre o *burnout* (IBM) apresentou um coeficiente de -0,26, com intervalo de confiança (IC) de 95%, variando de -0,44 a -0,08. O efeito indireto, mediado pela satisfação no trabalho, teve um coeficiente de -0,30, com IC de 95%, variando de -0,41 a -0,19. No modelo ajustado foi observado um valor de R² para a variável IBM igual a 45,2%. Ambos os efeitos, direto e indireto, foram estatisticamente significantes.

**Figura 1-:** Modelo teórico. Ribeirão Preto, SP, Brasil, 2021

*PES = *Practice Environment Scale*; ^†^IBM = Inventário de *Burnout* de Maslach

## Discussão

O presente estudo teve por objetivo analisar, à luz de um modelo teórico, a relação entre a percepção dos profissionais de enfermagem em unidades de terapia intensiva sobre o ambiente de prática, os níveis de *burnout* e a satisfação no trabalho.

O período de coleta de dados coincidiu com a segunda onda da pandemia da COVID-19 no Brasil. Naquele momento, o país passava por flutuações significativas nos números de casos; os óbitos eram expressivos; a vacinação de profissionais de saúde estava em estágios iniciais e a disseminação de variantes do vírus levantava preocupações adicionais sobre a transmissibilidade e a eficácia das vacinas^(23)^. É importante observar que esse contexto e as condições de trabalho daquele período podem ter influenciado na percepção dos profissionais sobre o ambiente de trabalho, os níveis de *burnout* e a satisfação no trabalho.

Os profissionais deste estudo foram, predominantemente, do sexo feminino, característica atribuída a uma construção histórica da categoria profissional da enfermagem^(24)^. A média de idade foi de 35,5 anos, e incluiu o tempo de experiência médio na função de 7,27 anos. Apesar de a jornada de trabalho na instituição ser de 30 horas semanais para os servidores públicos e de 36 horas para os contratos privados, os sujeitos informaram uma carga horária semanal média de 48,14 horas, que pode estar associada a outro vínculo empregatício. Quase metade dos profissionais de enfermagem estava envolvida em mais de um emprego. Essa situação pode ser uma resposta à necessidade de equilibrar demandas financeiras e a busca por estabilidade profissional. O duplo vínculo pode apresentar desafios em termos de esgotamento, disponibilidade de tempo pessoal e saúde geral, afetando negativamente tanto o equilíbrio físico quanto o emocional dos profissionais^(25)^.

A distribuição dos profissionais de enfermagem nas unidades de terapia intensiva representou a necessidade do período, quanto ao redirecionamento e à contração de pessoal dedicado ao tratamento intensivo da COVID-19. A mudança exigiu da enfermagem uma rápida adaptação e atualização de habilidades, uma vez que o cuidado dos pacientes apresentava desafios específicos e requerimentos de proteção adicionais devido à natureza contagiosa do vírus^(1,25)^. Apesar de o ambiente de prática analisado ter passado por tais adequações geradas pela pandemia, os profissionais de enfermagem reportaram uma percepção favorável do ambiente de prática, assim como em outros estudos^(14,26)^.

Quanto à classificação do nível de *burnout*, a maioria dos profissionais apresentou baixa exaustão emocional, elevada realização pessoal e moderada despersonalização. Em conjunto, os resultados do IBM indicam um quadro complexo. Apesar de a maioria dos profissionais relatar baixa exaustão emocional, não se pode desconsiderar a proporção de participantes com moderada a elevada exaustão emocional, o que sugere que a crise sanitária pode ter intensificado as demandas emocionais sobre os profissionais, possivelmente devido ao aumento do volume de trabalho, à exposição a situações de maior estresse e ao enfrentamento de desafios desconhecidos^(3,27-28)^.

Além disso, o nível de despersonalização reportado pode ser atribuído às situações adversas, como a necessidade de distanciamento e protocolos de segurança, as quais podem ter levado a um afastamento emocional entre os profissionais e os pacientes. Por outro lado, os profissionais que reportaram sentimento de realização pessoal podem ser um indicativo de resiliência e senso de propósito que eles encontraram ao lidar com a crise, devido ao reconhecimento da importância de seu trabalho no cenário pandêmico^(29)^.

No que diz respeito à satisfação no trabalho, o resultado reflete que os indivíduos estão satisfeitos em relação às suas atividades laborais, ou seja, apresentam uma percepção de que suas expectativas, necessidades e objetivos são atendidos no contexto do trabalho. Estudo que avaliou a relação entre percepção do ambiente de prática de enfermagem e a satisfação no trabalho entre enfermeiros de terapia intensiva resultou em ambiente de prática favorável, significativamente, correlacionado com a satisfação no trabalho, destacando ainda mais a importância de um ambiente saudável para resultados positivos para enfermagem^(14)^.

Quando comparada a exaustão emocional entre o sexo masculino e o feminino, as mulheres possuem maior nível de exaustão em relação aos homens. Algumas possíveis causas a serem consideradas incluem a existência de um segmento considerável de profissionais que possuem mais de um vínculo empregatício associado a atividades domésticas. Essa sobrecarga combinada pode intensificar significativamente os níveis de estresse e exaustão emocional entre as profissionais do sexo feminino^(25)^.

Verificou-se um menor nível de realização pessoal entre os enfermeiros. Esses, normalmente, exercem funções administrativas e de gerenciamento da equipe, tomam decisões clínicas e prestam menos cuidados diretos aos pacientes, situações essas que podem afetar a percepção da realização pessoal. Também entre os enfermeiros, pode haver uma expectativa maior quanto ao papel da liderança e relacionamento com a coordenação. Tais responsabilidades podem ser fonte extra de estresse e pressão, afetando a percepção da realização pessoal^(25,29)^.

O nível de exaustão emocional entre os turnos também apresentou diferença significante. A pontuação mais alta no turno da manhã indica que os profissionais de enfermagem apresentam maior exaustão emocional em comparação àqueles dos demais turnos. O turno da manhã geralmente apresenta maior demanda por coleta de exames, encaminhamentos para cirurgias e consultas multiprofissionais. Esse turno pode envolver uma série de tarefas e procedimentos que precisam ser realizados no início do dia. Essas demandas intensas e concentradas podem sobrecarregar os profissionais de enfermagem e contribuir para sentimentos de exaustão emocional^(27)^.

No que tange à adequação da equipe e de recursos, os profissionais do turno da noite apresentaram a menor pontuação e diferiram em relação aos profissionais do turno da tarde. Sabe-se que, durante a noite, o acesso ao serviço de apoio, como manutenção e informática, e o contato com as chefias e gerentes hospitalares é limitado, pois eles atuam em horário administrativo. Esses aspectos podem contribuir para uma percepção de recursos insuficientes no plantão noturno. Profissionais de saúde que operam em ambientes com recursos limitados ou inadequados frequentemente enfrentam uma sobrecarga de trabalho, o que aumenta o risco de gerar esgotamento emocional, redução da produtividade e insatisfação profissional. Esses problemas levam ao aumento do risco de erros e diminuem a qualidade dos cuidados prestados^(2,30)^.

No estudo das correlações, observou-se a forte correlação inversa entre exaustão emocional e satisfação no trabalho, que pode ser explicada pela natureza do ambiente de trabalho, ou seja, demandas intensas, carga emocional e estresse excessivo favorecem a diminuição na satisfação no trabalho e aumento da exaustão emocional, afetando o bem-estar e a motivação dos profissionais. Estudos demonstraram que melhores ambientes de trabalho foram correlacionados com menores taxas de insatisfação no trabalho e menor *burnout* ^(14,16)^.

Além disso, a correlação positiva de magnitude forte entre satisfação no trabalho, realização pessoal e as variáveis da PES podem ser interpretadas da seguinte forma: A satisfação no trabalho está ligada ao sentimento de cumprimento pessoal e à percepção de que o trabalho está de acordo com os objetivos e valores individuais. Profissionais satisfeitos tendem a estar mais engajados e motivados para fornecer um cuidado de maior qualidade aos pacientes. Um ambiente de trabalho onde os líderes oferecem suporte e promovem um clima positivo pode influenciar positivamente a satisfação e o desempenho dos profissionais. Relações positivas e colaborativas entre enfermeiros e médicos podem melhorar a comunicação, a coordenação e a eficácia da equipe^(31)^.

Por outro lado, houve também correlações moderadas entre exaustão emocional com todos os domínios da PES: os profissionais que sentem que não têm a oportunidade de influenciar as decisões sobre seu trabalho e o ambiente ao seu redor podem experienciar sentimentos de desgaste emocional. A falta de autonomia pode fazer com que os profissionais se sintam desvalorizados e desmotivados^(32)^. Adicionalmente, se os profissionais não sentirem que estão recebendo apoio adequado por parte da organização, isso pode aumentar o nível de exaustão emocional. A falta de comunicação, falta de suporte emocional e pouca clareza sobre as políticas da instituição contribuem para esse cenário^(31)^. Quando os recursos necessários para realizar o trabalho de forma adequada são escassos, os profissionais também podem se sentir exaustos^(11)^.

Outro aspecto importante é a correlação inversa de magnitude moderada entre satisfação no trabalho e despersonalização. Isso mostra que, quando técnicos de enfermagem e enfermeiros estão satisfeitos com seu trabalho, há uma tendência de que a despersonalização seja menor. Profissionais satisfeitos geralmente têm maior empatia e conexão com os pacientes, o que pode ajudar a evitar a despersonalização, um fenômeno em que os profissionais tratam os pacientes de maneira impessoal^(14)^.

Acerca da correlação positiva moderada entre a participação dos enfermeiros nos assuntos hospitalares e a disponibilidade de recursos adequados, quando os colaboradores da enfermagem se percebem valorizados e ouvidos em relação aos assuntos hospitalares, sua satisfação aumenta. Isso pode estar associado a um maior senso de importância e influência no ambiente de trabalho. Esse sentimento de valorização é reforçado quando os profissionais de enfermagem percebem ter recursos suficientes à disposição, resultando em maior satisfação no trabalho^(9,11)^.

Considerando as correlações identificadas neste estudo e o modelo teórico proposto, confirmou-se a relação entre as variáveis do ambiente de prática e o *burnout*. Essa relação se mantém presente, independentemente da inclusão da variável satisfação no trabalho no modelo. A constatação de uma mediação parcial significa que a relação entre ambiente de prática e *burnout* é explicada, tanto direta quanto indiretamente, pela satisfação no trabalho, ou seja, existem outros fatores, além da satisfação, que também podem mediar a forma como o ambiente de trabalho afeta o *burnout*. Um ambiente de prática favorável, com menor nível de *burnout*, engloba a disponibilidade de recursos adequados, sistemas de apoio efetivos, comunicação fluida entre as equipes e oportunidades contínuas de aprendizado, o que, por sua vez, viabiliza a aplicação de práticas em estudos científicos, estimula a colaboração interdisciplinar e fomenta a atenção segura ao paciente^(14,30)^.

No sentido oposto, trabalhar com equipamentos inadequados ou obsoletos e a falta de recursos essenciais podem aumentar a pressão sobre os profissionais de saúde, levando ao estresse, à exaustão emocional e a uma atitude insensível em relação ao trabalho e aos pacientes. Isso pode levar a cuidados de saúde inadequados e afetar a segurança do paciente^(5,30)^.

Na pandemia da COVID-19 houve inúmeros relatos e observações de alta carga de trabalho em hospitais. Esse estado de exaustão e a demanda extenuante colocaram os profissionais de saúde em um risco significativo de desenvolverem esgotamento físico e mental. Muitos deles enfrentaram situações extremamente complicadas, lidando com um número crescente de pacientes, falta de recursos adequados e ameaça constante de infecção. Por esse motivo, a discussão sobre a carga de trabalho está intimamente ligada à avaliação da suficiência e eficácia dos recursos humanos dentro de um ambiente de prática de enfermagem^(2)^.

Outro ponto de discussão nessa relação é a ausência de suporte da administração e dos gestores, que recebeu a menor pontuação entre as subescalas da PES avaliadas neste estudo. A falta de suporte pode contribuir com o *burnout*, pois pode fazer com que os profissionais se sintam desvalorizados. A escassez de *feedback*, reconhecimento e oportunidades de crescimento pode levar a uma sensação de estagnação, contribuindo para baixa realização pessoal, cultura de trabalho negativa e o aumento da rotatividade^(11)^.

No que tange aos sentimentos de despersonalização e de baixa realização pessoal, eles também podem ser associados ao relacionamento entre a enfermagem e médicos. Em ambiente de trabalho hostil, onde há rivalidade e falta de respeito, esperam-se atitudes conflituosas e uma desconexão com o trabalho e colegas. Isso prejudica a colaboração da equipe, a eficácia do tratamento e a satisfação do paciente, ou seja, as relações colegiais entre a enfermagem e os médicos são fundamentais para garantir um ambiente de saúde seguro e colaborativo. Essa troca de conhecimentos pode levar a melhores decisões de tratamento e atenção às necessidades dos pacientes^(2,27,30)^.

De forma geral, o ambiente de prática pode ter um efeito direto e profundo no esgotamento dos profissionais de saúde. A compreensão dessas relações é fundamental para criar estratégias eficazes a fim de prevenir e tratar o *burnout*. As intervenções podem incluir a melhoria da comunicação dentro da equipe, o fornecimento adequado de recursos, o ajuste da carga de trabalho e a promoção de uma cultura organizacional saudável.

Dentre as limitações deste estudo, destaca-se a dificuldade na coleta de dados em meio à crise sanitária. Os dados foram coletados de forma remota, não tendo sido possível acompanhar e ter o controle do número de leitos e de profissionais que sofreram oscilações influenciadas pelas ondas da pandemia.

## Conclusão

Os profissionais de enfermagem reportaram percepção favorável do ambiente de prática em todas as subescalas da PES. Acerca do *burnout*, a maior parte reportou baixa exaustão emocional, elevada realização pessoal e moderada despersonalização, além de estar satisfeita com o trabalho. O estudo demonstrou, por meio do modelo teórico, que ações de melhorias no ambiente de prática podem diminuir o nível de *burnout*, tanto de forma direta, quanto pelo aumento da satisfação no trabalho.

No entanto, a satisfação no trabalho não explica completamente essa relação, sugerindo que existem outros fatores não examinados neste modelo que também podem influenciar o *burnout*. Os resultados destacam a complexidade do *burnout*, implicando que, mesmo em locais de trabalho que possuem ambiente de prática favorável e satisfatório, alguns colaboradores ainda enfrentam dificuldades relacionadas ao *burnout*.

Em termos práticos, recomendam-se, como estratégias suporte emocional, a comunicação aberta, o aprendizado contínuo e o acompanhamento dessas variáveis no período pós-pandemia, visando mitigar os impactos negativos da pandemia e favorecer um ambiente de trabalho mais salubre e equilibrado.

Os resultados deste estudo trazem implicações relevantes para a prática profissional em enfermagem, especialmente no contexto das unidades de terapia intensiva. A identificação da satisfação no trabalho como mediadora parcial entre o ambiente de prática e o *burnout* destaca a importância de estratégias institucionais voltadas à qualificação do ambiente organizacional, como a valorização do profissional, o fortalecimento da liderança, a melhoria das condições de trabalho e a promoção de relações colaborativas entre as equipes. Tais ações podem contribuir diretamente para a redução do *burnout* e a promoção da saúde ocupacional dos profissionais. O estudo também propõe um modelo teórico que amplia a compreensão das interações entre variáveis organizacionais e psicossociais no contexto da enfermagem, podendo subsidiar novas investigações. Recomenda-se que estudos futuros explorem outros potenciais mediadores e moderadores dessa relação, incluindo aspectos individuais, organizacionais e culturais, além de pesquisas longitudinais que permitam o acompanhamento das variáveis ao longo do tempo e em diferentes cenários assistenciais.

## Data Availability

Os conjuntos de dados relacionados a este artigo estarão disponíveis mediante solicitação ao autor correspondente.

# Translation: Entorno de práctica y *burnout* en unidades de cuidados intensivos: ¿la satisfacción laboral actúa como mediadora de esta relación?*

**DOI:** 10.1590/1518-8345.7659.4712

**Authors:** Jussara Aparecida da Silva Furlan, Edinêis de Brito Guirardello

**Affiliations:** 1 Universidade Estadual de Campinas, Faculdade de Enfermagem, Campinas, SP, Brasil

**Keywords:** Enfermería, Agotamiento Profesional, Ambiente de Instituciones de Salud, Satisfacción en el Trabajo, Unidades de Cuidados Intensivos, Agotamiento Emocional

## Abstract

analizar, con base en un modelo teórico, la relación entre la percepción de los profesionales de enfermería de las unidades de cuidados intensivos sobre el entorno de práctica, el nivel de *burnout* y la satisfacción laboral.

estudio transversal y correlacional realizado con 114 profesionales de enfermería de un hospital universitario del noroeste del estado de São Paulo. Se utilizaron la ficha de caracterización personal y profesional, la *Practice Environment Scale*, el Inventario de *Burnout* de Maslach y la subescala de satisfacción laboral del *Safety Attitudes Questionnaire*. Se efectuaron análisis descriptivos, pruebas de comparación (prueba *t* de Student para muestras independientes, Mann-Whitney, ANOVA y Kruskal-Wallis), correlaciones (Pearson o Spearman) y modelado de ecuaciones estructurales para verificar el efecto mediador.

los profesionales reportaron un entorno favorable para la práctica, satisfacción laboral, bajo nivel de agotamiento emocional, elevado nivel de realización personal y nivel moderado de despersonalización. Se constató que la satisfacción laboral ejerció un efecto mediador parcial entre el entorno de práctica y el *burnout*.

las acciones orientadas a mejorar el entorno de práctica pueden reducir el *burnout* de forma directa o mediante el aumento de la satisfacción laboral. Comprender estas relaciones es esencial para promover un entorno favorable, minimizar el *burnout* y aumentar la satisfacción en el trabajo.

## Destacados

**(1)** El estudio confirma la relación entre el entorno de práctica y el *burnout*. **(2)** La satisfacción laboral media parcialmente la relación entre el entorno y el *burnout*. **(3)** Mejorar el entorno reduce el *burnout* tanto de forma directa como mediante el aumento de la satisfacción. **(4)** El modelo teórico ayuda a los gestores a identificar intervenciones específicas.

## Introducción

El entorno de práctica de la enfermería se compone de las características organizacionales del ambiente laboral que facilitan o dificultan la práctica de la profesión^(1)^. En las Unidades de Cuidados Intensivos (UCI), dicho entorno es altamente especializado y multidimensional, con el objetivo principal de proporcionar cuidados intensivos a pacientes gravemente enfermos. Los profesionales de enfermería que trabajan en estas unidades realizan actividades orientadas a la estabilización y recuperación de los pacientes, y también están expuestos a impactos emocionales derivados de las exigencias y características de este lugar de trabajo^(2-3)^.

En los últimos años, las actividades realizadas por la enfermería cobraron protagonismo debido a su papel en la atención de pacientes con COVID-19. Las UCI fueron los entornos con mayor demanda asistencial, experimentando una expansión del número de camas y un aumento de la necesidad de recursos humanos y materiales. Este escenario obligó a los profesionales de enfermería a desempeñar sus funciones en medio de la escasez de recursos y la sobrecarga laboral^(2)^, además de presenciar innumerables muertes, el contagio de compañeros, el propio contagio y el temor constante de llevar el virus a casa^(4)^, configurando un ambiente de práctica propicio al deterioro de la salud profesional^(3,5)^.

Ante este contexto de sobrecarga y adversidades durante la pandemia, resulta fundamental comprender qué elementos del entorno laboral contribuyen a la salud y al desempeño del equipo de enfermería. Se considera un entorno laboral favorable aquel que permite al equipo de enfermería ejercer sus actividades con autonomía, dispone de recursos humanos y materiales adecuados, cuenta con gestores que mantienen una comunicación abierta y ofrecen oportunidades de desarrollo profesional. En cambio, un entorno desfavorable se caracteriza por la escasez de suministros, la sobrecarga de trabajo, la ausencia de apoyo del liderazgo, la falta de colaboración entre los equipos de salud, la insatisfacción y el *burnout* entre los profesionales^(6-7)^.

Las condiciones de salud asociadas directamente al entorno laboral están ampliamente descritas en la literatura, entre ellas el *burnout*, un problema frecuente entre los profesionales de enfermería. Este síndrome se asocia a la presión excesiva en el trabajo, lo que se traduce en agotamiento emocional, despersonalización y disminución de la realización personal^(8)^. Los estudios señalan altas tasas de *burnout* entre enfermeros que trabajan en UCI^(3,8)^, con repercusiones negativas tanto en la seguridad del paciente^(9)^ como en la calidad de la atención^(10-11)^.

Una revisión sistemática que analizó 85 estudios con 288.581 enfermeros de 32 países identificó una prevalencia media de burnout del 30,7% (± 9,7%). El *burnout* se asoció con desenlaces negativos, como un peor clima de seguridad, mayor incidencia de caídas de pacientes, errores de medicación, cuidados omitidos, menor calidad de la atención e insatisfacción de los pacientes, independientemente de la edad, sexo, experiencia profesional o localización geográfica^(9)^.

La relación entre *burnout* y satisfacción laboral ha sido analizada entre profesionales de enfermería que trabajan en unidades de cuidados intensivos. El *burnout* y la satisfacción en el trabajo presentan una relación inversamente proporcional. Una revisión sistemática reciente, con 18 estudios y un total de 3168 enfermeros de UCI, identificó una prevalencia global de burnout del 37,5% y reveló una correlación inversa significativa entre burnout y satisfacción laboral, evidenciando que los profesionales más satisfechos muestran menor riesgo de desarrollar el síndrome^(12)^. Los altos niveles de agotamiento emocional y despersonalización se asocian a una disminución de la satisfacción profesional, impactando directamente la motivación, el compromiso y la calidad de la asistencia prestada. Por otro lado, los profesionales satisfechos tienden a presentar menor nivel de *burnout*, mayor resiliencia frente a las adversidades del entorno y mejor percepción de su propio desempeño^(13)^.

En este contexto, la satisfacción laboral puede actuar como un factor protector frente al agotamiento emocional y el desgaste profesional. La misma metaanálisis señaló que los profesionales satisfechos muestran mayor resiliencia, mejor desempeño y menores índices de ausentismo y rotación^(12)^. La satisfacción del equipo de enfermería no se refiere solamente al bienestar individual de sus miembros y a la percepción de su vida profesional, sino también a la calidad y la salud del entorno laboral. La gestión ineficiente del entorno de práctica, asociada a altos niveles de agotamiento emocional, se ha relacionado con un aumento de la insatisfacción laboral entre los profesionales de enfermería^(9)^.

Considerando esta relación, al analizar conjuntamente el entorno de práctica, el *burnout* y la satisfacción laboral, se observa que los entornos positivos se correlacionan con menores niveles de *burnout y* menores niveles de insatisfacción profesional^(7,14)^. El análisis integrado de estos aspectos permite comprender la contribución del entorno de práctica a los resultados de enfermería y a los desenlaces de los pacientes, y brinda a los gestores la posibilidad de identificar los puntos que requieren mejoras^(1)^. Esto significa que un entorno considerado favorable para la práctica de la enfermería puede generar repercusiones positivas para los pacientes, los profesionales y las organizaciones^(15)^.

A pesar del creciente volumen de investigaciones sobre el entorno de práctica de la enfermería, aún son escasos los estudios que investigan de forma integrada la relación entre entorno de práctica, *burnout* y satisfacción laboral de los profesionales de enfermería. La literatura muestra avances en la comprensión individual de estos elementos; sin embargo, existe una laguna en lo que respecta al análisis conjunto de estos factores y a la propuesta de modelos teóricos que expliquen sus interacciones.

Ante la importancia del entorno de práctica en el agotamiento profesional y en la satisfacción de los profesionales de enfermería en las UCI, este estudio parte de la siguiente pregunta orientadora: ¿la satisfacción laboral medía la relación entre el entorno de práctica y el burnout en profesionales de enfermería que trabajan en UCI? Por lo tanto, el objetivo de este estudio fue analizar, con base en un modelo teórico, la relación entre la percepción de los profesionales de enfermería de las unidades de cuidados intensivos acerca del entorno de práctica, el nivel de burnout y la satisfacción laboral.

## Método

### Tipo

Estudio transversal y correlacional que siguió las recomendaciones del *Strengthening the Reporting of Observational Studies in Epidemiology* (STROBE).

### Lugar

Estudio realizado en unidades de cuidados intensivos de adultos de un hospital universitario de la ciudad de Ribeirão Preto, SP, Brasil. Este hospital fue referencia para los casos críticos de COVID-19 en su área de cobertura, por lo que amplió el número de camas de cuidados intensivos. Al inicio de la recolección de datos contaba con 84 camas intensivas distribuidas en seis Unidades de Cuidados Intensivos; la recolección se realizó en las seis UCI, todas ellas incluidas en el estudio.

El vínculo de los profesionales que actúan en esta institución comprende diferentes modalidades de contratación. Los colaboradores pueden ser contratados mediante contratos temporales, según las necesidades específicas de la institución, o bajo el régimen de la Consolidación de las Leyes del Trabajo por medio de una Fundación. Además, la mayoría del personal está constituida por concursados. La jornada de trabajo se organiza en tres turnos: mañana y tarde, con duración de 6 horas cada uno, y noche, con un periodo de 12 horas. Adicionalmente, los colaboradores en funciones administrativas cumplen un horario regular de 8 horas diarias.

### Período

La recolección de datos se realizó entre mayo y noviembre de 2021.

### Población y muestra

La población estuvo compuesta por 202 profesionales de enfermería. El cálculo se efectuó utilizando la metodología para estimar el tamaño muestral para una proporción. En el cálculo se empleó una proporción p igual a 0,50, que representa la máxima variabilidad de la distribución binomial, generando así una estimación con el mayor tamaño muestral practicable. La muestra correspondió a 133 profesionales y participaron en el estudio 114 sujetos seleccionados por conveniencia.

### Criterios de selección

Se incluyeron los profesionales que actuaban en la atención directa a los pacientes, técnicos de enfermería y enfermeros asistenciales, así como los enfermeros que desempeñaban funciones de gestión en las UCI. Se excluyeron los profesionales que, en el momento de la recolección de datos, se encontraban ausentes por licencia u otras formas de ausencia legal.

### Variables e instrumentos

Las variables edad (en años cumplidos), sexo (femenino, masculino, prefiero no declarar), función (técnico de enfermería o enfermero), tiempo de experiencia profesional (en años), turno de trabajo (diurno, nocturno y otros), unidad de trabajo, presencia de más de un vínculo laboral (sí o no) y carga horaria semanal (en horas) se recolectaron mediante una ficha de caracterización personal y profesional.

Para la recolección de datos relacionados con el entorno laboral, *burnout* y satisfacción se utilizaron las versiones brasileñas de los siguientes instrumentos, previamente validados para la población objetivo: *Practice Environment Scale* (PES)^(16)^, Inventario de *Burnout* Maslach (IBM)^(17)^ y la subescala satisfacción en el trabajo del *Safety Attitudes Questionnaire* (SAQ) – *Short Form* 2006^(18)^.

La PES^(16)^ tiene por finalidad evaluar la percepción de los profesionales sobre el entorno de práctica. La escala contiene 24 ítems distribuidos en cinco subescalas: 1) participación de los enfermeros en la discusión de asuntos hospitalarios; 2) fundamentos de enfermería orientados a la calidad de la atención; 3) habilidad, liderazgo y apoyo de los coordinadores/supervisores de enfermería a los enfermeros/equipo de enfermería; 4) adecuación de la dotación y de los recursos; 5) relaciones colegiadas entre profesionales de enfermería y médicos. Las opciones de respuesta se presentan mediante una escala Likert de 4 puntos: 1 (totalmente en desacuerdo), 2 (en desacuerdo), 3 (de acuerdo) y 4 (totalmente de acuerdo). Puntajes iguales o superiores a 2,5 en cuatro o cinco subescalas se clasifican como entornos favorables; puntajes iguales o superiores a 2,5 en dos o tres subescalas se consideran entornos mixtos; y resultados superiores a 2,5 en ninguna o solo en una subescala se evalúan como entornos desfavorables para la práctica^(1)^. Para el presente estudio, los valores de confiabilidad, evaluados mediante el alfa de Cronbach, fueron de 0,80 participación de los enfermeros en la discusión de asuntos hospitalarios, 0,79 fundamentos de enfermería orientados a la calidad de la atención, 0,85 habilidad, liderazgo y apoyo de los coordinadores/supervisores de enfermería a los enfermeros/equipo de enfermería, 0,71 adecuación de la dotación y de los recursos y 0,76 relaciones colegiadas entre profesionales de enfermería y médicos.

El Inventario de *Burnout* Maslach^(17)^ se empleó para evaluar el nivel de *burnout* de los profesionales. Está compuesto por 22 ítems distribuidos en tres dimensiones: agotamiento emocional, despersonalización y realización personal, evaluadas en una escala Likert. La puntuación del agotamiento emocional varía de 9 a 45 puntos y la de despersonalización de 5 a 25 puntos; en ambas, puntuaciones más altas indican mayor nivel de *burnout*. La dimensión realización personal varía de 8 a 40 puntos, siendo inversamente proporcional al nivel de *burnout*; es decir, cuanto menor la puntuación, mayor el nivel de *burnout* percibido. Los puntajes obtenidos en cada dimensión se dividieron en terciles, clasificados como bajo, moderado o alto. Para este estudio, la confiabilidad, evaluada mediante el alfa de Cronbach, fue de 0,89 para agotamiento emocional, 0,63 para despersonalización y 0,77 para disminución de la realización personal. Para la aplicación del IBM se adquirió la licencia a *MindGarden* ^©^.

La variable satisfacción en el trabajo, derivada de la subescala satisfacción en el trabajo del *Safety Attitudes Questionnaire* ^(18)^, incluye cinco ítems evaluados en una escala Likert de cinco puntos. Puntajes medios iguales o superiores a 75 puntos se clasificaron como satisfacción en el trabajo^(18)^. Para este estudio, la confiabilidad de esta subescala, medida mediante el alfa de Cronbach, fue de 0,80.

### Recolección de datos

Los datos se recolectaron mediante un formulario en línea. El enlace fue enviado por los gestores de enfermería, una sola vez, al correo electrónico corporativo y se puso a disposición mediante código QR en carteles ubicados en los puestos de enfermería, con información sobre el público objetivo.

### Tratamiento y análisis de los datos

Se realizó análisis descriptivo de las variables cualitativas mediante frecuencias absolutas y relativas, mientras que para las variables cuantitativas se emplearon medidas de posición y dispersión. En el análisis comparativo se utilizaron las pruebas t de Student para muestras independientes o Mann-Whitney, *Analysis of Variance* (ANOVA) seguida de la prueba de Tukey, y Kruskal-Wallis seguida de la prueba de Dunn. Para los análisis de correlación se emplearon los coeficientes de Pearson o Spearman^(19)^ e interpretaciones de correlación débil (0,10 a 0,29), moderada (0,30 a 0,49) y fuerte (≥ 0,50)^(19)^. La elección de pruebas paramétricas o no paramétricas se basó en la evaluación de la distribución de los datos mediante *boxplots*, con valoración de *outliers*, y mediante la prueba de Shapiro-Wilk. Estos análisis se realizaron con *Statistical Analysis System* (SAS) versión 9.4 y *Statistical Package for the Social Sciences* (SPSS) versión 23(20).

Para investigar la presencia de un efecto mediador de la satisfacción laboral en la relación entre el entorno y el *burnout* (variable dependiente), se utilizó la modelización de ecuaciones estructurales con el método *Partial Least Squares* (PLS)^(21)^ en el *software Smart* PLS 3.3.5(22). Se consideró mediación completa cuando el efecto indirecto fue significativo y el directo no; mediación parcial cuando ambos fueron significativos; y ausencia de mediación cuando el efecto indirecto no fue significativo^(21)^.

### Aspectos éticos

Este estudio fue aprobado por el Comité de Ética en Investigación, dictamen n.º 3.717.632. La participación fue voluntaria, tras la aceptación del Consentimiento Informado, disponible en formato digital.

## Resultados

La muestra estuvo constituida por 114 profesionales de enfermería, de los cuales 35,1% (n = 40) eran enfermeros y 64,9% (n = 74) técnicos de enfermería. De ellos, 78,1% (n = 89) pertenecían al sexo femenino, con una edad media de 35,5 años (desviación estándar [DE] = 7,91). En cuanto al lugar de trabajo, el 54,4% (n = 64) informó trabajar en las UCI COVID y el 45,6% (n = 52) en las UCI clínicas y quirúrgicas.

Respecto a la experiencia profesional, señalaron una media de 7,27 años (DE = 5,85) en la función y una media de 3,73 años (DE = 4,58) en el sector. Además, el 46,49% (n = 53) tenía otro vínculo laboral y la carga horaria semanal media fue de 48,14 horas. La mayoría del personal trabajaba en el turno nocturno (45,6%, n = 52).

En la Tabla 1 se presentan los resultados de las subescalas de la *Practice Environment Scale* y de la subescala de satisfacción laboral. Se observa que los profesionales reportaron una percepción favorable del entorno, con medias superiores a 2,5 que oscilaron entre 2,64 y 3,09, así como satisfacción con el trabajo (M = 79,69).

**Tabla 1-:** Percepción de los profesionales de enfermería según las subescalas de la *Practice Environment Scale* y satisfacción laboral. Ribeirão Preto, SP, Brasil, 2021

**Variables**	**Media**	**Desviación estándar**
Participación de los enfermeros en la discusión de asuntos hospitalarios	2,64	0,73
Fundamentos de enfermería orientados a la calidad de la atención	2,83	0,59
Adecuación del personal y de los recursos	3,01	0,61
Capacidad, liderazgo y apoyo de los coordinadores/supervisores de enfermería a los enfermeros/equipo de enfermería	3,06	0,72
Relaciones colegiadas entre profesionales de enfermería y médicos	3,09	0,60
Satisfacción laboral	79,69	17,52

Para la clasificación del nivel de *burnout* se consideró la distribución de los valores en terciles, bajo, medio y alto, para cada dimensión del *burnout* (Tabla 2). Se destaca que el nivel de agotamiento emocional fue bajo (≤ 22; 37,7%), el de realización personal fue elevado (≤ 29; 38,6%) y el de despersonalización fue moderado (> 7,63 y ≤ 11; 39,5%).

**Tabla 2-:** Nivel de *burnout* reportado por los profesionales de enfermería. Ribeirão Preto, SP, Brasil, 2021

**Dimensiones del *burnout***	**Bajo**	n*	**%**	**Moderado**	n*	**%**	**Elevado**	n*	**%**
Agotamiento emocional	≤ 22	43	37,7	> 22 e ≤ 27,3	33	29	> 27,3	38	33,3
Realización personal	> 33	35	30,7	> 29 e ≤ 33	35	30,7	≤ 29	44	38,6
Despersonalización	≤ 7,63	38	33,3	> 7,63 e ≤ 11	45	39,5	> 11	31	27,1

*n = Muestra

En el análisis comparativo de las variables del estudio, incluidas las características personales y profesionales, la percepción del entorno de práctica, el *burnout* y la satisfacción laboral, se consolidaron aquellas que mostraron diferencias estadísticamente significativas, como agotamiento emocional, realización personal, turno de trabajo y adecuación de la dotación y de los recursos (Tabla 3).

**Tabla 3-:** Comparación de la percepción de los profesionales de enfermería respecto a las dimensiones del IBM* y las subescalas de la PES^†^ en relación con el sexo, la función y el turno. Ribeirão Preto, SP, Brasil, 2021

**Variables**			**Media**	**Desviación estándar**	**p-valor**
IBM* - Agotamiento Emocional	Sexo	Masculino	22,58	7,77	0,0318 ^‡^
		Femenino	25,84	6,15	
IBM* - Realización Personal	Función	Técnico	31,84	4,17	0,0078 ^‡^
		Enfermero	29,63	4,14	
IBM* - Agotamiento Emocional	Turno	Mañana	28,07	6,78	0,0147 ^§||^
		Tarde	20,88	6,27	
		Noche	25,04	6,49	
		Administrativo	26,27	6,13	
PES ^†^ - Adecuación del equipo y de recursos	Turno	Mañana	2,89	0,82	0,0141 ^¶^ **
		Tarde	3,45	0,47	
		Noche	2,92	0,55	
		Administrativo	2,96	0,58	

*IBM = Inventario de *Burnout* de Maslach; ^†^PES = *Practice Environment Scale*; ^‡^Valor p obtenido mediante la prueba t de Student para muestras independientes; ^§^Valor p obtenido mediante la prueba ANOVA; ^||^Posprueba de Tukey (diferencia significativa entre Mañana × Tarde); ^¶^Valor p obtenido mediante la prueba de Kruskal-Wallis; **Posprueba de Dunn (diferencia significativa Tarde × Noche)

Las mujeres presentaron mayores niveles de agotamiento en comparación con los hombres (p = 0,0318); los profesionales del turno de la mañana reportaron mayor nivel de agotamiento emocional en relación con los de los turnos de la tarde y la noche (p = 0,0147); y los técnicos de enfermería presentaron mayor nivel de realización personal en comparación con los enfermeros (p = 0,0078). Además, la percepción de adecuación de la dotación y los recursos fue mayor en el turno de la tarde en comparación con el nocturno (p = 0,0141).

En la Tabla 4 se presentan las correlaciones entre las dimensiones del *burnout*, las subescalas del entorno de práctica y la satisfacción laboral. Se observa la existencia de una correlación de gran magnitud entre la satisfacción laboral y las dimensiones agotamiento emocional (r = -0,54) y realización personal (r = 0,58), así como una correlación de gran magnitud entre la satisfacción laboral y las subescalas Fundamentos de enfermería orientados a la calidad de la atención (r = 0,57), Habilidad, liderazgo y apoyo de los gestores de enfermería a los enfermeros/equipo de enfermería (r = 0,54) y Relaciones colegiadas entre profesionales de enfermería y médicos (r = 0,55).

**Tabla 4-:** Correlación entre las dimensiones del IBM*, las subescalas de la PES^†^ y la subescala de satisfacción laboral del SAQ^‡^. Ribeirão Preto, SP, Brasil, 2021

**Variables**	IBM*- Agotamiento Emocional	IBM*- Realización Personal	IBM* - Despersonalización	PES ^†^ - Participación de los enfermeros en la discusión de asuntos hospitalarios	PES ^†^ - Fundamentos de enfermería orientados a la calidad de la atención	PES ^†^ - Habilidad, liderazgo y apoyo de los gestores de enfermería a los enfermeros/equipo de enfermería	PES ^†^ - Adecuación del personal y de los recursos	PES ^†^ - Relaciones colegiadas entre profesionales de enfermería y médicos
PES ^†^ - Participación de los enfermeros en la discusión de asuntos hospitalarios	-0,33 ^§||^	0,37 ^§||^	-0,15 ^||^					
PES ^†^ - Fundamentos de enfermería orientados a la calidad de la atención	-0,42 ^§¶^	0,46 ^§¶^	-0,24 ^§^					
PES ^†^ - Habilidad, liderazgo y apoyo de los gestores de enfermería a los enfermeros/equipo de enfermería	-0,46 ^§||^	0,42 ^§||^	-0,30 ^§||^					
PES ^†^ - Adecuación del personal y de los recursos	-0,43 ^§||^	0,26 ^§||^	-0,13 ^||^					
PES ^†^ - Relaciones colegiadas entre profesionales de enfermería y médicos	-0,47 ^§||^	0,47 ^§||^	-0,38 ^§||^					
SAQ ^‡^ - Satisfacción laboral	-0,54 ^§||^	0,58 ^§||^	-0,35 ^§||^	0,47 ^§||^	0,57 ^§||^	0,54 ^§||^	0,37 ^§||^	0,55 ^§||^

*IBM = Inventario de *Burnout* de Maslach; ^†^PES = *Practice Environment Scale*; ^‡^SAQ = *Safety Attitudes Questionnaire*; ^§^p-valor < 0,05; ^||^Coeficiente de correlación de *Spearman*. Intensidad de la correlación (valores positivos y negativos): fuerte ≥ 0,50; moderada: 0,30 a 0,49; débil: 0,1 a 0,29; ^¶^Coeficiente de correlación de Pearson

A partir de la revisión de la literatura y de las correlaciones encontradas, se propuso un modelo teórico, presentado en la Figura 1, en el que el entorno de práctica se definió como variable independiente, el *burnout* como variable dependiente y la satisfacción laboral se clasificó como variable mediadora. El efecto directo del entorno de práctica (PES) sobre el *burnout* (IBM) mostró un coeficiente de -0,26, con intervalo de confianza (IC) del 95 %, que varió de -0,44 a -0,08. El efecto indirecto, mediado por la satisfacción laboral, tuvo un coeficiente de -0,30, con IC del 95 %, que osciló entre -0,41 y -0,19. En el modelo ajustado se observó un valor de R² para la variable IBM de 45,2 %. Ambos efectos, directo e indirecto, fueron estadísticamente significativos.

**Figura 1-:** Modelo teórico. Ribeirão Preto, SP, Brasil, 2021

*PES = *Practice Environment Scale*; ^†^IBM = Inventario de *Burnout* de Maslach

## Discusión

El presente estudio tuvo como objetivo analizar, a la luz de un modelo teórico, la relación entre la percepción de los profesionales de enfermería en unidades de cuidados intensivos acerca del entorno de práctica, los niveles de *burnout* y la satisfacción laboral.

El periodo de recogida de datos coincidió con la segunda ola de la pandemia de COVID-19 en Brasil; en ese momento, el país experimentaba fluctuaciones significativas en el número de casos, los decesos eran elevados, la vacunación de los profesionales sanitarios se encontraba en fases iniciales y la propagación de variantes del virus suscitaba preocupaciones adicionales sobre la transmisibilidad y la eficacia de las vacunas^(23)^. Es importante señalar que este contexto y las condiciones de trabajo de ese periodo pudieron influir en la percepción de los profesionales sobre el entorno laboral, los niveles de *burnout* y la satisfacción en el trabajo.

Los participantes de este estudio fueron predominantemente mujeres, característica atribuida a la construcción histórica de esta categoría profesional^(24)^. La edad media fue de 35,5 años e incluyó una experiencia media de 7,27 años en la función. A pesar de que la jornada laboral en esta institución es de 30 horas semanales para los servidores públicos y de 36 horas para los contratos privados, los sujetos informaron una carga horaria semanal media de 48,14 horas, lo que puede estar asociado a otro vínculo laboral; casi la mitad de los profesionales de enfermería desempeña más de un empleo. Esta situación puede constituir una respuesta a la necesidad de equilibrar las demandas financieras y la búsqueda de estabilidad profesional, pero el doble vínculo puede plantear desafíos en términos de agotamiento, disponibilidad de tiempo personal y salud general, afectando negativamente tanto el equilibrio físico como el emocional de los profesionales^(25)^.

La distribución de los profesionales de enfermería en las unidades de cuidados intensivos reflejó la exigencia del periodo en cuanto al redireccionamiento y la contratación de personal dedicado al tratamiento intensivo de la COVID-19. Este cambio exigió a la enfermería una rápida adaptación y actualización de habilidades, ya que la atención de estos pacientes presentaba desafíos específicos y requerimientos adicionales de protección debido a la naturaleza contagiosa del virus^(1,25)^. Aun con las adecuaciones generadas por la pandemia, los profesionales de enfermería informaron una percepción favorable del entorno de práctica, resultado coincidente con otros estudios^(14,26)^.

En cuanto a la clasificación del nivel de *burnout*, la mayoría presentó bajo agotamiento emocional, alta realización personal y despersonalización moderada. En conjunto, los resultados del IBM revelan un panorama complejo: aunque la mayoría declaró bajo agotamiento emocional, no puede ignorarse la proporción con agotamiento moderado o alto, lo que sugiere que la crisis sanitaria pudo intensificar las demandas emocionales debido al aumento del volumen de trabajo, la exposición a situaciones de mayor estrés y el enfrentamiento de desafíos desconocidos^(3,27-28)^.

Además, el nivel de despersonalización reportado puede atribuirse a las situaciones adversas, como la necesidad de distanciamiento y los protocolos de seguridad, que pudieron propiciar un alejamiento emocional entre profesionales y pacientes. Por otro lado, el sentimiento de realización personal comunicado por algunos profesionales puede indicar resiliencia y sentido de propósito al afrontar la crisis, debido al reconocimiento de la importancia de su labor en el escenario pandémico^(29)^.

Respecto a la satisfacción laboral, el resultado refleja que los individuos están satisfechos con sus actividades laborales, es decir, perciben que sus expectativas, necesidades y objetivos se satisfacen en el contexto del trabajo. Un estudio que evaluó la relación entre la percepción del entorno de práctica de enfermería y la satisfacción laboral en enfermeros de cuidados intensivos mostró que un entorno de práctica favorable se correlacionó de forma significativa con la satisfacción laboral, lo que subraya aún más la importancia de un entorno saludable para resultados positivos en enfermería^(14)^.

Al comparar el agotamiento emocional entre los sexos, se constató que las mujeres presentan un nivel mayor de agotamiento respecto a los hombres. Entre las posibles causas se incluye la existencia de un segmento considerable con más de un vínculo laboral asociado a las tareas domésticas. Esta sobrecarga combinada puede intensificar significativamente los niveles de estrés y agotamiento emocional entre las profesionales femeninas^(25)^.

Se observó un nivel menor de realización personal entre los enfermeros. Estos suelen desempeñar funciones administrativas, gestionar el equipo, tomar decisiones clínicas y proporcionar menos cuidados directos a los pacientes, situaciones que pueden afectar la percepción de realización personal. Además, entre los enfermeros puede existir una expectativa mayor en cuanto al papel de liderazgo y la relación con la coordinación. Estas responsabilidades pueden constituir una fuente adicional de estrés y presión, repercutiendo en la percepción de realización personal^(25,29)^.

El nivel de agotamiento emocional entre los turnos también presentó una diferencia significativa. La puntuación más alta en el turno de la mañana indica que los profesionales de enfermería experimentan mayor agotamiento emocional en comparación con los demás turnos. La mañana suele implicar mayor demanda de toma de muestras, derivaciones para cirugías y consultas multiprofesionales. Este turno puede agrupar una serie de tareas y procedimientos que deben realizarse al inicio del día; dichas demandas intensas y concentradas pueden sobrecargar a los profesionales de enfermería y contribuir a sentimientos de agotamiento emocional^(27)^.

En lo que respecta a la adecuación del personal y de los recursos, los profesionales del turno nocturno presentaron la puntuación más baja y difirieron de los del turno de la tarde. Se sabe que, durante la noche, el acceso a servicios de apoyo como mantenimiento e informática, así como el contacto con las jefaturas y directivos hospitalarios, es limitado, pues estos trabajan en horario administrativo. Estos aspectos pueden contribuir a una percepción de recursos insuficientes durante el turno nocturno. Los profesionales de salud que operan en entornos con recursos limitados o inadecuados a menudo enfrentan una sobrecarga de trabajo, lo que incrementa el riesgo de agotamiento emocional, reduce la productividad y genera insatisfacción profesional. Tales problemas aumentan el riesgo de errores y disminuyen la calidad de la atención prestada^(2,30)^.

En el estudio de las correlaciones se observó una fuerte correlación inversa entre agotamiento emocional y satisfacción laboral, lo que puede explicarse por la naturaleza del entorno de trabajo: demandas intensas, carga emocional y estrés excesivo favorecen la disminución de la satisfacción laboral y el aumento del agotamiento emocional, afectando el bienestar y la motivación profesionales. Estudios han demostrado que mejores entornos de trabajo se correlacionan con menores tasas de insatisfacción laboral y *burnout* ^(14,16)^.

Además, la correlación positiva de magnitud fuerte entre satisfacción laboral, realización personal y las variables de la PES puede interpretarse del siguiente modo: la satisfacción laboral está vinculada al sentimiento de logro personal y a la percepción de que el trabajo se alinea con los objetivos y valores individuales. Los profesionales satisfechos tienden a estar más comprometidos y motivados para proporcionar cuidados de mayor calidad a los pacientes. Un entorno laboral en el que los líderes ofrecen apoyo y promueven un clima positivo puede influir favorablemente en la satisfacción y el rendimiento de los profesionales. Las relaciones positivas y colaborativas entre enfermeros y médicos pueden mejorar la comunicación, la coordinación y la eficacia del equipo^(31)^.

Por otra parte, se hallaron correlaciones moderadas entre el agotamiento emocional y todos los dominios de la PES: los profesionales que sienten no tener oportunidad de influir en las decisiones sobre su trabajo y el entorno que les rodea pueden experimentar desgaste emocional. La falta de autonomía puede hacer que se perciban desvalorizados y desmotivados^(32)^. Asimismo, si perciben que no reciben apoyo adecuado por parte de la organización, el nivel de agotamiento emocional aumenta; la ausencia de comunicación, de soporte emocional y de claridad en las políticas institucionales contribuye a este escenario^(31)^. Cuando los recursos necesarios para realizar el trabajo de forma adecuada son escasos, los profesionales también pueden sentirse exhaustos^(11)^.

Otro aspecto relevante es la correlación inversa de magnitud moderada entre la satisfacción laboral y la despersonalización: cuando los técnicos de enfermería y los enfermeros están satisfechos con su trabajo, la tendencia a la despersonalización disminuye. Los profesionales satisfechos suelen mostrar mayor empatía y conexión con los pacientes, lo que ayuda a evitar la despersonalización, fenómeno en el que los pacientes son tratados de manera impersonal^(14)^.

Respecto a la correlación positiva moderada entre la participación de los enfermeros en los asuntos hospitalarios y la disponibilidad de recursos adecuados, cuando el personal de enfermería se percibe valorado y escuchado, su satisfacción se incrementa. Esto puede asociarse a un mayor sentido de importancia e influencia en el entorno laboral, reforzado cuando perciben disponer de recursos suficientes, lo que resulta en mayor satisfacción^(9,11)^.

Considerando las correlaciones identificadas y el modelo teórico propuesto, se confirmó la relación entre las variables del entorno de práctica y el *burnout*. Esta relación se mantiene presente independientemente de la inclusión de la satisfacción laboral en el modelo; la mediación parcial indica que la relación entre entorno de práctica y *burnout* se explica tanto de forma directa como indirecta mediante la satisfacción laboral, es decir, existen otros factores que también pueden mediar la manera en que el entorno de trabajo afecta al *burnout*. Un entorno favorable, con menor nivel de *burnout*, implica disponibilidad de recursos adecuados, sistemas de apoyo efectivos, comunicación fluida entre equipos y oportunidades continuas de aprendizaje, lo que facilita la aplicación de prácticas basadas en evidencia, estimula la colaboración interdisciplinar y fomenta la atención segura al paciente^(14,30)^.

En sentido opuesto, trabajar con equipos inadecuados u obsoletos, o con falta de recursos esenciales, incrementa la presión sobre los profesionales, conduciendo al estrés, al agotamiento emocional y a una actitud insensible hacia el trabajo y los pacientes. Esto puede provocar una atención deficiente y comprometer la seguridad del paciente^(5,30)^.

Durante la pandemia de COVID-19 se reportó una elevada carga de trabajo en los hospitales; este estado de agotamiento y demanda extenuante situó a los profesionales de salud en riesgo significativo de desarrollar desgaste físico y mental, enfrentando un número creciente de pacientes, falta de recursos adecuados y amenaza constante de infección. Por ello, discutir la carga de trabajo está estrechamente vinculado a la evaluación de la suficiencia y eficacia de los recursos humanos dentro del entorno de práctica de enfermería^(2)^.

Otro punto en esta relación es la ausencia de apoyo de la administración y de los gestores, que obtuvo la puntuación más baja entre las subescalas de la PES evaluadas. La falta de apoyo puede contribuir al *burnout*, pues hace que los profesionales se sientan desvalorizados. La escasez de *feedback*, reconocimiento y oportunidades de crecimiento conduce a una sensación de estancamiento, contribuyendo a la baja realización personal, a una cultura laboral negativa y al aumento de la rotación^(11)^.

En lo que respecta al sentimiento de despersonalización y la baja realización personal, estos también pueden vincularse a la relación entre enfermería y médicos. En un entorno laboral hostil, donde predominan la rivalidad y la falta de respeto, se esperan actitudes conflictivas y una desconexión con el trabajo y los colegas. Ello perjudica la colaboración del equipo, la eficacia del tratamiento y la satisfacción del paciente; por tanto, las relaciones colegiadas entre enfermería y médicos son fundamentales para garantizar un entorno sanitario seguro y colaborativo. Este intercambio de conocimientos puede conducir a mejores decisiones terapéuticas y a una mayor atención a las necesidades de los pacientes^(2,27,30)^.

En términos generales, el entorno de práctica puede ejercer un efecto directo y profundo en el agotamiento de los profesionales de la salud. Comprender estas relaciones es esencial para crear estrategias eficaces que prevengan y aborden el *burnout*. Las intervenciones pueden incluir la mejora de la comunicación dentro del equipo, el suministro adecuado de recursos, el ajuste de la carga de trabajo y la promoción de una cultura organizacional saludable.

Entre las limitaciones de este estudio se destaca la dificultad de recolectar datos en medio de la crisis sanitaria, realizada de forma remota, lo que impidió acompañar y controlar las oscilaciones en el número de camas y de profesionales influidas por las oleadas de la pandemia.

## Conclusión

Los profesionales de enfermería informaron una percepción favorable del entorno de práctica en todas las subescalas de la PES; respecto al *burnout*, la mayoría reportó bajo agotamiento emocional, alta realización personal y despersonalización moderada, además de sentirse satisfechos con el trabajo. El estudio demostró, mediante el modelo teórico, que las acciones para mejorar el entorno de práctica pueden disminuir el nivel de *burnout* tanto de forma directa como mediante el aumento de la satisfacción laboral.

No obstante, la satisfacción laboral no explica completamente esta relación, lo que sugiere que existen otros factores no examinados en este modelo que también pueden influir en el burnout. Los resultados subrayan la complejidad del *burnout*, pues incluso en lugares de trabajo con entornos favorables y satisfactorios, algunos colaboradores siguen enfrentando dificultades relacionadas con este síndrome.

En la práctica, se recomiendan estrategias de apoyo emocional, comunicación abierta, aprendizaje continuo y seguimiento de estas variables en el periodo pospandémico, a fin de mitigar los impactos negativos de la pandemia y favorecer un entorno laboral más saludable y equilibrado.

Los resultados de este estudio aportan implicaciones relevantes para la práctica profesional de enfermería, especialmente en el contexto de las unidades de cuidados intensivos. La identificación de la satisfacción laboral como mediadora parcial entre el entorno de práctica y el *burnout* resalta la importancia de estrategias institucionales orientadas a la cualificación del entorno organizacional, como la valorización del profesional, el fortalecimiento del liderazgo, la mejora de las condiciones laborales y la promoción de relaciones colaborativas entre los equipos. Tales acciones pueden contribuir directamente a reducir el *burnout* y promover la salud ocupacional de los profesionales. El estudio también propone un modelo teórico que amplía la comprensión de las interacciones entre variables organizacionales y psicosociales en el contexto de la enfermería, pudiendo sustentar nuevas investigaciones. Se recomienda que futuros estudios exploren otros posibles mediadores y moderadores de esta relación, incluidos aspectos individuales, organizacionales y culturales, además de investigaciones longitudinales que permitan el seguimiento de las variables a lo largo del tiempo y en diferentes escenarios asistenciales.

## Data Availability

Los conjuntos de datos relacionados con este artículo estarán disponibles previa solicitud al autor correspondiente.
